# Benzo[*b*]tellurophenes as a Potential Histone H3 Lysine 9 Demethylase (KDM4) Inhibitor

**DOI:** 10.3390/ijms20235908

**Published:** 2019-11-25

**Authors:** Yoon-Jung Kim, Dong Hoon Lee, Yong-Sung Choi, Jin-Hyun Jeong, So Hee Kwon

**Affiliations:** 1 College of Pharmacy, Yonsei Institute of Pharmaceutical Sciences, Yonsei University, 85 Songdogwahak-ro, Yeonsu-gu, Incheon 21983, Korea; sunshine333@sookmyung.ac.kr (Y.-J.K.); tci30@naver.com (D.H.L.); uriys2@gmail.com (Y.-S.C.); 2 Department of Integrated OMICS for Biomedical Science, Yonsei University, Seoul 03720, Korea

**Keywords:** tellurium, benzo[*b*]tellurophene, benzo[*b*]selenophene, KDM4 inhibitor, histone H3K9 demethylases

## Abstract

Gene expression and tumor growth can be regulated by methylation levels of lysine residues on histones, which are controlled by histone lysine demethylases (KDMs). Series of benzo[*b*]tellurophene and benzo[*b*]selenophene compounds were designed and synthesized and they were evaluated for histone H3 lysine 9 demethylase (KDM4) inhibitory activity. Among the carbamates, alcohol and aromatic derivatives, tert-butyl benzo[*b*]tellurophen-2-ylmethylcarbamate (compound 1c) revealed KDM4 specific inhibitory activity in cervical cancer HeLa cells, whereas the corresponding selenium or oxygen substitute compounds did not display any inhibitory activity toward KDM4. Compound 1c also induced cell death in cervical and colon cancer but not in normal cells. Thus, compound 1c, a novel inhibitor of KDM4, constitutes a potential therapeutic and research tool against cancer.

## 1. Introduction

Benzofuran and benzothiophene are famous molecular frameworks that are oxygen- or sulfur-containing heterocycles. These frames provide useful ligands for more than one type of receptor or enzyme target via structural modifications, called privileged structure [1,2,3,4]. The benzofuran structure is found in amiodarone (calcium, potassium, and sodium channels; antiarrhythmic), dronedarone (calcium, potassium, and sodium channels; antiarrhythmic), vilazodone (serotonin receptor; antidepressant), and many opioid drugs (opioid receptors; analgesic) [1,3,4]. Benzothiophene is part of raloxifene (estrogen receptor, osteoporosis in postmenopausal women), zileuton (5-lipoxygenase, asthma), sertaconazole (14α-demethylase, antifungal), and benocyclidine *N*-methyl-d-aspartate receptor (NMDAR), psychostimulant) [2]. These privileged structures serve as structural motif templates, which play important roles in medicinal chemistry.

Selenium and tellurium belong to the calcogens: The same periodic table group as oxygen and sulfur. They have been considered nonessential and toxic elements in the past. Recently, selenium has been found in the human body as selenoproteins like glutathione peroxidase (GPx), and organoselenium compounds including ebselen have been synthesized and evaluated for biological functions [5,6]. Our group reported that the substitution of oxygen in flavonoid structures with selenium improves their antioxidant effect in vitro and in vivo [7]. Organotellurium compounds also are of interest, and several groups have reported their biological activities [8,9,10,11,12]. A vitamin E derivative containing tellurium mimicking the action of GPx shows catalytic antioxidant activity [13]. Selenium- and tellurium-containing molecules are likely to be new privileged structures that could widen the molecular universe to find novel medicines for many unconquered targets.

Interestingly, ebselen and some selenium compounds have shown inhibitory activity toward histone H3 lysine 9 demethylase (KDM4) by ejection of structural Zn(II) [14]. Histone methylation performs an important role in the regulation of eukaryotic cellular systems such as transcriptional regulation, chromatin organization, and gene expression [15,16,17]. Methylation of specific lysine residues is regulated by histone methyltransferases (HMTs) and histone demethylases (KDMs) [15]. Lysine-specific demethylases, KDM proteins in the JmjC-domain-containing demethyalse (JMJD) family function as a 2-oxoglutarate-dependent Fe(II) oxygenase and catalyzes hydroxylation reactions [16]. Recent data indicate that abnormal expression of KDM4 proteins can promote the progression of cancer, including breast cancer, prostate cancer, and lymphomas [18], and thus have emerged as potential therapeutic targets. For example, KDM4A–C are overexpressed at both the protein and mRNA levels in breast tumors, and their overexpression contributes to breast tumorigenesis by stimulating estrogen receptor α (ERα) activity [18,19,20,21]. Overexpression of KDM4C increases the expression of the mouse double minute 2 (MDM2) oncogene in a manner dependent on its demethylase activity, leading to a decrease of tumor suppressor p53 target genes including proapoptotic genes [20] and to promotion of breast tumor growth [21]. Thus, KDM4 inhibitors represent potential selective cancer therapeutic agents for cancer treatment [22,23,24,25]. Moreover, ebselen has a selenium-containing benzo-fused five-membered heterocycle, similar to that of benzofuran and benzothiophene, as its core structure. We set out to identify structurally unique, selective inhibitor of KDM4 in cancer cells, and based on the evidence above, we were inspired to synthesize benzo[*b*]selenophene and benzo[*b*]tellurophene compounds and evaluate their activity against KDMs. Especially, benzo[*b*]tellurophenes having carbamate or alcohol groups are novel compounds that have never been synthesized to our knowledge.

**Figure 1:**
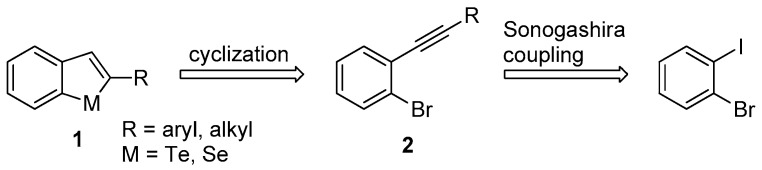
Representative structure and the synthetic strategy of benzo[*b*]tellurophene and benzo[*b*]selenophene (compound 1).

Figure 1 depicts α-substituted benzo[*b*]tellurophenes and benzo[*b*]selenophenes (1) and our synthetic strategy for the preparation of these compounds. We expected to synthesize compound 1 by the ring cyclization of 2 with NaHTe/NaHSe [26], which was obtained from the commercially available compound 1-bromo-2-iodobenzene, by a Sonogashira reaction. Herein, we prepared novel benzo[*b*]tellurophenes and benzo[*b*]selenophenes (1) and evaluated their inhibitory activity on KDM4.

## 2. Results and Discussion

### 2.1. Chemistry

**Scheme 1:**
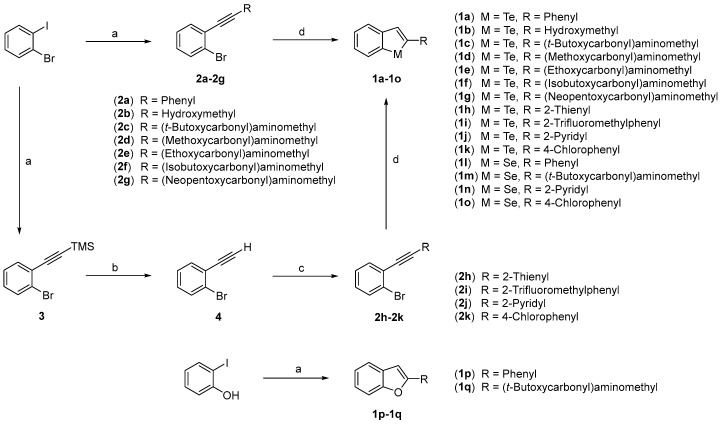
Reagents and conditions: (**a**) HC≡CR, PdCl_2_(PPh_3_)_2_, CuI, piperidine, toluene, 45 °C, 4 h; (**b**) K_2_CO_3_, MeOH, RT, 1 h; (**c**) I-R, PdCl_2_(PPh_3_)_2_, CuI, piperidine, toluene, 45 °C, 4 h; (**d**) NaHM (M = Te, Se), DMF, 100 °C, o/n.

Preparation of the benzo[*b*]tellurophene, benzo[*b*]selenophene, and benzo[*b*]furanderivatives is outlined in Scheme 1. Compounds 2a–g and 3 were easily obtained by Sonogashira reaction of 1-bromo-2-iodobenzene with corresponding terminal alkynes. Compound 4 was obtained by reductive removal of the trimethylsilyl (TMS) group of 3 under K_2_CO_3_ and MeOH conditions. The reaction of 1-bromo-2-ethynylbenzene (4) with various iodoaryl compounds yielded compounds 2h–2k by Sonogashira reaction. Treatment of compounds 2a–k with sodium hydrogen telluride (selenide), generated in situ from tellurium (selenium) powder and sodium borohydride in dimethylformamide (DMF) at 100 °C, resulted in ring closure, affording the desired benzo[*b*]tellurophenes and benzo[*b*]selenophenes (1a–o). Benzo[*b*]furans (1p,q) were also synthesized from 2-iodophenol with corresponding terminal alkynes under Sonogashira reaction conditions to compare their biological activity with benzo[*b*]tellurophene and benzo[*b*]selenophene compounds. Benzo[*b*]tellurophene, benzo[*b*]selenophene, and benzo[*b*]furan derivatives with carbamates, alcohol, and aromatic moieties were designed and synthesized. Compounds 1a,lp have the most similar structure with ebselen which has a simple phenyl group conjugated with a benzo-fused five-membered heterocyle. This phenyl group was replaced with carbamate (1c–g,m,q), alcohol (1b), or heteroaryl (1h,j,n) moieties, which can form a polar interaction with internal metals or the hydrophilic residues of proteins. Other compounds (1i,k,o) contained phenyl groups with halogens, which also can interact with polar atoms. All the prepared compounds are listed in Table 1 (Supplementary Materials).

### 2.2. Biological Evaluation

#### 2.2.1. Effects of Compounds on Histone Methylation in cervical cancer HeLa Cells

**Figure 2:**
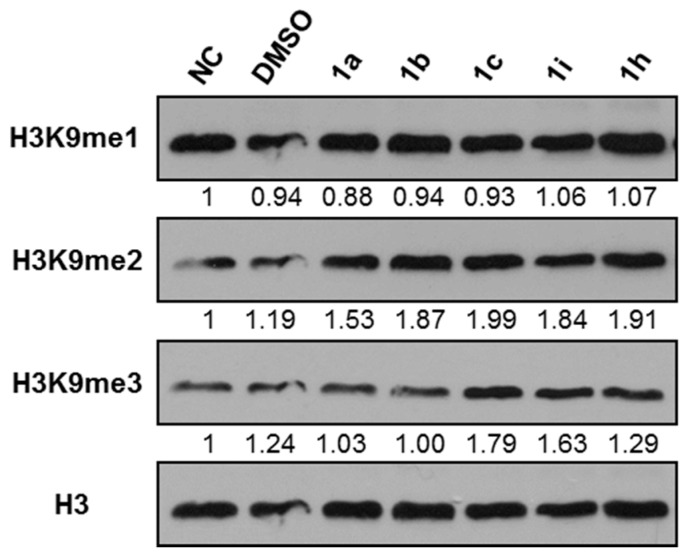
Effects of various compounds on histone H3 lysine 9 (H3K9) methylation. Cervical cancer HeLa cells were treated with negative control (NC), 0.1% DMSO, or 10 μM of the indicated compounds. After 24 h, histones extracted from HeLa cells were resolved on a 15% SDS-polyacrylamide gel. Immunoblotting was performed with anti-pan histone H3, anti-H3K9me3, anti-H3K9me2, and anti-H3K3me1 antibodies. Levels of H3K9me were semi‑quantified relative to H3, with the levels in the NC groups set at 1. Histone H3 is shown as an equal loading control.

All the prepared compounds were tested for inhibition of KDM4 in HeLa cells by immunoblot analysis of acid-extracted histones using antibodies specific for methylated histone H3 lysine 9 (H3K9me) (a substrate of KDM4) and in vitro histone demethylase assay. Human cervical cancer HeLa cells were cultured with 10 μM of 15 different benzo[*b*]tellurophen, benzo[*b*]selenophen, or benzo[*b*]furan derivatives for 24 h (Table 1) and we analyzed the methylated levels of H3K9 by immunoblotting. Levels of trimethylated H3K9 (H3K9me3) were semi‑quantified relative to histone H3; the levels in the negative control (NC) groups were set to 1, and a >1.5-fold increase in H3K9me3 level was considered to indicate a significant inhibitory effect for KDM4. Based on immunoblot analysis, Benzo[*b*]tellurophene compounds 1c and 1i inhibited KDM4 activity as identified by increased H3K9me3 level compared to NC even when considering the effects of DMSO (Figure 2 and Table 1). The carbamate compound 1c especially showed much significant inhibition, whereas the corresponding selenium compound 1m and oxygen compound 1q did not display any inhibitory activity toward KDM4 (Table 1). Although the exact mechanisms of action of compound 1c or 1i are not clear, our present results show that the benzo[*b*]tellurophene structure is critical to inhibition of KDM4 activity.

To date, two possible mechanisms of KDM inhibition are known. Inhibitors adhere to the binding site of oxo-glutarate or the zinc binding region of KDMs. Both of these inhibition mechanisms are mediated by nonbonding interactions with the metal (Fe or Zn) coexisting with KDMs. Oxygen, selenium, and tellurium have six valence electrons, but tellurium has the lowest electronegativity of these. For this reason, tellurium is more easily coordinated with metals. Nitrogen or oxygen atoms of carbamate groups also can interact with metals. Tellurium and the carbamate moiety of compound 1c might interact with metals by bi- or tri-dentate modes. Thus, they could be stronger inhibitors of KDM4 than 1a. From the result that other carbamate derivatives (1d–g) show weak inhibition activity, we conclude that the *tertiary*-butyl group of 1c might be the most effective structure for inhibiting KDM4. A partial charge between carbon- and a halogen (δ^+^-δ^−^) has the potential to interact with polar atoms near the molecule. The KDM4 inhibition activity of compound 1i may be due to an additional interaction between the ortho-trifluoromethyl substitution of the phenyl group and polar residues of the protein. However, the compound with a meta-chloro substituted phenyl ring (1k) showed no different activity than the compound with a simple phenyl ring (1a). Compounds (1b,h,j), which have heteroatoms at a three-bond distance from tellurium that could interact with metal, did not sufficiently inhibit KDM4. Therefore, we have selected 1c as the most potent candidate through the above considerations.

**Figure 3:**
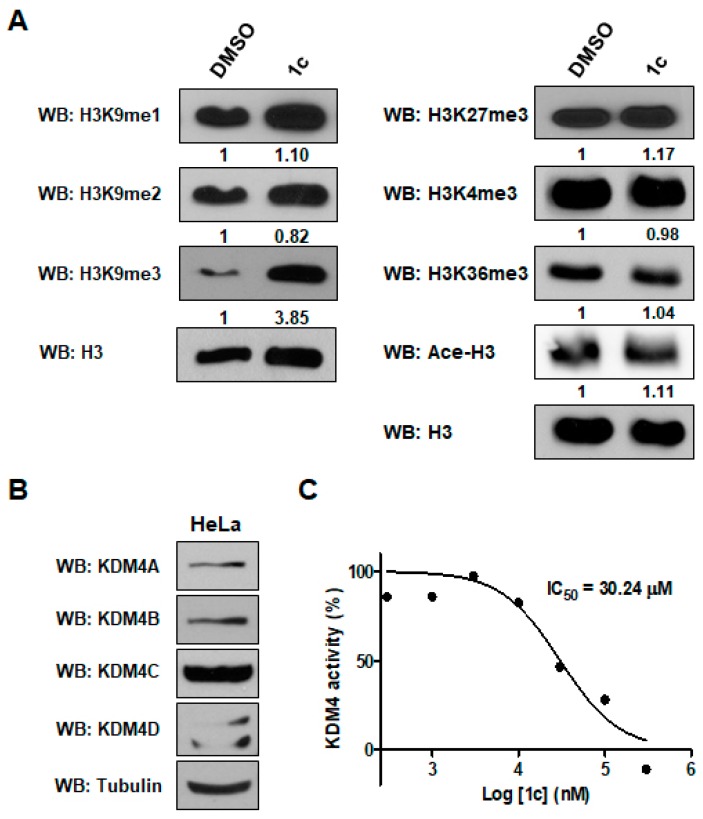
Compound 1c inhibits demethylase activity of KDM4. (**A**) HeLa cells were treated with NC, 0.1% DMSO (control) or 10 μM of 1**c**, as indicated. After 24 h, histones extracted from HeLa cells were resolved on a 15% SDS-polyacrylamide gel. Immunoblotting was performed with anti-pan histone H3, anti-H3K9me3, anti-H3K9me2, anti-H3K3me1, anti-H3K27me3, anti-H3K4me3, anti-H3K36me3, and anti- ace-H3 antibodies. Levels of H3K9me and ace-H3 were semi‑quantified relative to H3 and the levels in the DMSO groups were set at 1. Histone H3 is shown as an equal loading control. (**B**) Expression levels of KDM4 proteins in HeLa cells. (**C**) Inhibitory data (IC_50_) of KDM4 demethylation activity by 1c. Formaldehyde dehydrogenase (FDH)-coupled demethylase assay was used for IC_50_ determination. Data are expressed as mean ± SD from three independent experiments.

To further confirm the potency and specificity of compound 1c as a KDM4 inhibitor, we tested other histone modifications as well as histone H3 lysine 9 (H3K9) methylation by immunoblot analysis. Treatment of HeLa cells with 1c (10 μM) led to a substantial increase in global H3K9me3 levels, which signifies the accumulation of methylated lysine by inhibition of the demethylation process of KDM4 (Figure 3A). We also tested the expression levels of four different KDM4 (KDM4A–D) proteins in HeLa cells (Figure 3B). Levels of monomethylated and dimethylated H3K9 (H3K9me2/me1), which are specifically demethylated by KDM3, were not increased by 1c, allowing us to conclude that 1c is not an inhibitor of KDM3. Compound 1c did not influence the methylation of other lysine residues such as K27, K4, or K36 of histone H3. Because KDM6, KDM5, and KDM2/7 demethylate histones H3K27, H3K4, and H3K36, respectively, this result suggests that 1c does not inhibit KDM6, KDM5, or KDM2/7. In addition, 1c did not affect histone H3 acetylation by histone acetyltransferase (Ace-H3 in Figure 3A). This finding indicates that 1c can selectively inhibit KDM4 activity.

#### 2.2.2. KDM4 Inhibitory Assays

To directly monitor the potency of 1c against KDM4, we tested its effect on the demethylase activity of recombinant KDM4A on a H3K9me3 peptide using a formaldehyde dehydrogenase (FDH)-coupled assay. Consistent with immunoblotting, the compound 1c demonstrated concentration-dependent inhibition of KDM4 enzymatic activity with an IC_50_ of 30.24 ± 4.60 μM, as illustrated in Figure 3C. These results strongly imply that 1c can specifically inhibit histone H3 lysine 9 demethylase KDM4.

#### 2.2.3. Effects of 1c on Cytotoxicity of Human Cervical HeLa and Colon LoVo Cancer Cells

**Figure 4:**
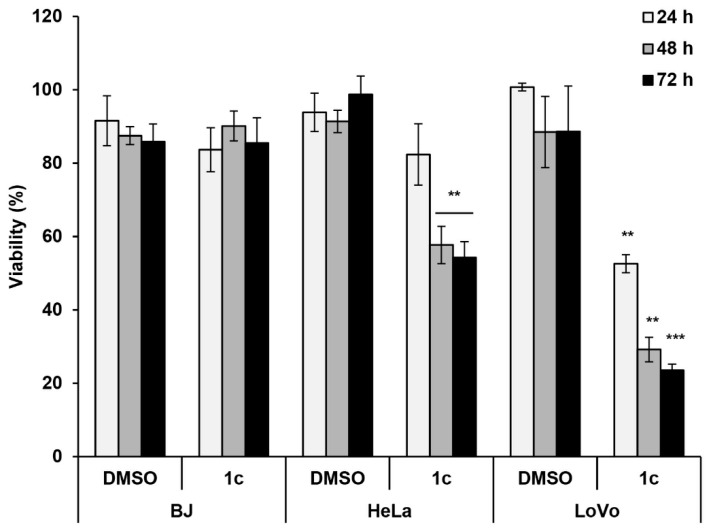
Effects of 1c on cell viability. BJ, HeLa, and LoVo cells were cultured with of 0.1% DMSO (control) or 10 μM 1c for 72 h, and MTT assays were performed to measure cell viability. Data are expressed as mean ± SD from three independent experiments. ** *p* < 0.01 and *** *p* < 0.001 vs. the DMSO control.

As KDM4 is deregulated in cancer, we examined the anticancer potential of 1c. To determine the specificity of 1c for cancer cells, we conducted cell viability assays in cervical cancer HeLa cells, colon cancer LoVo cells, and normal human foreskin fibroblast BJ cells using MTT assays. Compound 1c decreased the viability of HeLa and LoVo cells but not BJ cells (Figure 4). This finding suggests that KDM4-selective inhibition by compound 1c only induces cell death in transformed but not normal cells.

## 3. Materials and Methods

### 3.1. Chemistry

General Remarks. All reagents were purchased from commercial sources and used as received without purification. Reactions were monitored by thin-layer chromatography on 0.25 mm silica plate (F-254) visualizing with UV light (254 nm) and KMnO_4_ solution. Flash chromatography was performed using silica gel (230–400 mesh) with hexanes and EtOAc as eluent. ^1^H and ^13^C NMR spectra were recorded on Agilent 400 MHz NMR spectrometer. Chemical shifts (δ) are reported in parts per million (ppm), and coupling constants (*J*) are expressed in hertz (Hz). IR spectra were recorded on FT-IR using diamond attenuated total reflection (ATR) technique and were described as wavenumbers (cm^−1^). High resolution mass spectrometry (HRMS) were measured with electrospray ionization (ESI) and quadrupole time of flight (Q-TOF) mass analyzer. Physicochemical properties were generated by CS ChemProp. Cellular fluorescence was measured with multi-plate reader (Tecan, Männedorf, Switzerland) at 490 nm/520 nm. NMR spectra of compound 1a-1q is provided as supplementary materials.

#### 3.1.1. General Procedure for the Preparation of Compounds 2a, 2b, and 3

To a solution of acetylene compounds (3.6 mmol) in toluene (20 mL), piperidine (4 mL), 1-bromo-2-iodobenzene (1.27 g, 3.6 mmol), PdCl_2_(Ph_3_P)_2_ (127 mg, 0.2 mmol), and CuI (35 mg, 0.2 mmol) were added, which was then stirred at 45 °C for 4 hours. After the mixture was cool, water was added and the resulting mixture was extracted with methylene chloride. The combined organic layers were washed with water and brine, then dried over MgSO_4_, filtered, and concentrated in vacuo. The residue was purified by flash column chromatography using *n*-hexane: ethyl acetate = 5:1 as the eluent.

The 1-Bromo-2-phenylethynylbenzene (2a). Yield: 98%; IR (neat, cm^−1^) 3055, 2219; ^1^H NMR (400 MHz, CDCl_3_) δ 7.64–7.50 (m, 3H), 7.39–7.32 (m, 1H), 7.29 (t, *J* = 7.6 Hz, 1H), 7.17 (t, *J* = 7.6 Hz, 1H); ^13^C NMR (100 MHz, CDCl_3_) δ 133.2, 132.4, 131.7, 129.3, 128.6, 128.3, 127.0, 125.6, 125.4, 122.9, 93.9, 88.0.

The 3-(2-Bromophenyl)prop-2-yn-1-ol (2b). Yield: 61%; IR (neat, cm^−1^) 3324, 2232; ^1^H NMR (400 MHz, CDCl_3_) δ 7.56 (d, *J* = 8.0 Hz, 1H), 7.45 (d, *J* = 7.6 Hz, 1H), 7.24 (t, *J* = 7.6 Hz, 1H), 7.15 (t, *J* = 7.6 Hz, 1H), 4.54 (s, 2H), 2.34 (s, OH); ^13^C NMR (100 MHz, CDCl_3_) δ 133.6, 132.4, 129.6, 127.0, 125.4, 124.7, 91.9, 84.1, 51.6.

The 2-Bromophenylethynyltrimethylsilane (3). Yield: 92%; IR (neat, cm^−1^) 2958, 2161, 1464, 1248; ^1^H NMR (400 MHz, CDCl_3_) δ 7.56 (dd, *J* = 8.0, 1.2 Hz, 1H), 7.48 (dd, *J* = 7.6, 1.6 Hz, 1H), 7.23 (td, *J* = 7.6, 1.2 Hz, 1H), 7.15 (td, *J* = 7.6, 1.6 Hz, 1H), 0.28 (s, 9H); ^13^C NMR (100 MHz, CDCl_3_) δ 133.6, 132.3, 129.5, 126.9, 125.7, 125.2, 103.0, 99.6, 0.2.

#### 3.1.2. General Procedure for the Preparation of Compound 2c–2g

Chlorocarbonate derivatives (4.4 mmol, 2c: di-tert-butyl dicarbonate) were slowly added to a solution of propagyl amine (200 mg, 3.6 mmol) in piperidine (4 mL) at 0 °C. The reaction mixture was stirred at room temperature for 3 hours. Toluene (20 mL), 1-bromo-2-iodobenzene (1.27 g, 3.6 mmol), PdCl_2_(Ph_3_P)_2_ (127 mg, 0.2 mmol), and CuI (35 mg, 0.2 mmol) were added to the resulting mixture, which was then stirred at 45 °C for 4 hours. After the mixture was cooled, water was added and the resulting mixture was extracted with methylene chloride. The combined organic layers were washed with water and brine, then dried over MgSO_4_, filtered, and concentrated in vacuo. The residue was purified by flash column chromatography using *n*-hexane: ethyl acetate = 5:1 as the eluent.

The tert-Butyl 3-(2-bromophenyl)prop-2-ynylcarbamate (2c). Yield: 92%; IR (neat, cm^−1^) 3345, 2969, 1677, 1518; ^1^H NMR (400 MHz, CDCl_3_) δ 7.56 (dd, *J* = 8.0, 0.2 Hz, 1H), 7.44 (dd, *J* = 7.6, 1.6 Hz, 1H), 7.24 (td, *J* = 7.6, 1.0 Hz, 1H), 7.15 (td, *J* = 7.8, 1.6 Hz, 1H), 4.86 (NH, 1H), 4.20 (d, *J* = 4.4 Hz, 2H), 1.47 (s, 9H); ^13^C NMR (100 MHz, CDCl_3_) δ 155.3, 133.5, 132.3, 129.5, 127.0, 125.5, 124.8, 90.1, 81.7, 80.0, 31.3, 28.4.

Methyl 3-(2-bromophenyl)prop-2-ynylcarbamate (2d). Yield: 93 %; IR (neat, cm^−1^) 3319, 2952, 1698; ^1^H NMR (400 MHz, CDCl3) δ 7.56 (d, *J* = 8.0 Hz, 1H), 7.44 (d, *J* = 7.6 Hz, 1H), 7.25 (t, *J* = 8.0 Hz, 1H), 7.16 (t, *J* = 7.6 Hz, 1H), 5.05 (s, NH), 4.27 (d, *J* = 4.8 Hz, 2H), 3.72 (s, 3H); ^13^C NMR (100 MHz, CDCl3) δ 156.5, 133.5, 132.4, 129.6, 127.0, 125.5, 124.6, 89.7, 81.9, 52.5, 31.8.

Ethyl 3-(2-bromophenyl)prop-2-ynylcarbamate (2e). Yield: 95%; IR (neat, cm^−1^) 3317, 2979, 1694, 1510, 1468; ^1^H NMR (400 MHz, CDCl_3_) 7.5 (d, *J* = 8.0 Hz, 1H), 7.43 (d, *J* = 7.6, 1.2 Hz, 1H), 7.22 (t, *J* = 7.6 Hz, 1H), 7.14 (t, *J* = 8.0, 1.6 Hz, 1H), 5.26 (s, NH), 4.26 (d, *J* = 4.8 Hz, 2H), 4.17 (d, *J* = 6.8 Hz, 2H), 1.25 (t, *J* = 6.8 Hz, 3H); ^13^C NMR (100 MHz, CDCl_3_) δ 156.2, 133.5, 132.3, 129.5, 127.0, 125.4, 124.7, 89.9, 81.7, 61.2, 31.6, 14.6.

Isobutyl 3-(2-bromophenyl)prop-2-ynylcarbamate (2f). Yield: 92%; IR (neat, cm^−1^) 3323, 2959, 1697, 1509, 1467; ^1^H NMR (400 MHz, CDCl_3_) δ 7.54 (d, *J* = 8.0 Hz, 1H), 7.43 (d, *J* = 7.6 Hz, 1H), 7.22 (t, *J* = 7.6 Hz, 1H), 7.14 (t, *J* = 7.6 Hz, 1H), 5.29 (s, NH), 4.26 (d, *J* = 4.8 Hz, 2H), 3.89 (d, *J* = 6.6 Hz, 2H), 1.91 (sep, *J* = 6.6 Hz, 1H), 0.92 (d, *J* = 6.6 Hz, 6H); ^13^C NMR (100 MHz, CDCl_3_) δ 156.4, 133.5, 132.3, 129.5, 126.9, 125.4, 124.7, 90.0, 81.3, 71.4, 31.7, 28.0, 19.0.

Neopentyl 3-(2-bromophenyl)prop-2-ynylcarbamate (2g). Yield: 97%; IR (neat, cm^−1^) 3303, 2957, 1694, 1515; ^1^H NMR (400 MHz, CDCl_3_) δ 7.57 (d, *J* = 8.0 Hz, 1H), 7.45 (d, *J* = 7.6 Hz, 1H), 7.24 (td, *J* = 7.6, 0.8 Hz, 1H), 7.17 (td, *J* = 8.0, 1.6 Hz, 1H), 4.97 (s, NH), 4.28 (d, *J* = 5.0 Hz, 2H), 3.81 (s, 2H), 0.94 (s, 9H); ^13^C NMR (100 MHz, CDCl_3_) δ 156.4, 133.5, 132.4, 129.6, 127.0, 125.5, 124.7, 89.8, 81.9, 74.7, 31.7, 31.5, 26.4.

#### 3.1.3. Procedure for the Preparation of Compound 4

The 1-Bromo-2-ethynylbenzene (4). K_2_CO_3_ (7.0 g, 51.01 mmol) was added to a solution of compound 3 (4.3 g, 17.00 mmol) in MeOH (20 mL). The reaction mixture was stirred at room temperature for 1 hour, then poured into water and extracted with ethyl acetate. The organic layers were washed with brine, dried over MgSO_4_, filtered, and concentrated in vacuo (2.9 g, 15.9 mmol). Yield: 94%; IR (neat, cm^−1^) 3289, 2111; ^1^H NMR (400 MHz, CDCl_3_) δ 7.59 (dd, *J* = 8.0, 1.1 Hz, 1H), 7.53 (dd, *J* = 7.6, 1.6 Hz, 1H), 7.27 (td, *J* = 7.6, 1.6 Hz, 1H), 7.20 (td, *J* = 7.6, 1.6 Hz, 1H), 3.38 (s, 1H); ^13^C NMR (100 MHz, CDCl_3_) δ 134.1, 132.5, 129.9, 126.9, 125.5, 124.3, 81.9, 81.8.

#### 3.1.4. General Procedure for the Preparation of Compound 2h–2k

Piperidine (2 mL), PdCl_2_(Ph_3_P)_2_ (35 mg, 0.05 mmol), and CuI (9.5 mg, 0.05 mmol) were added to a mixture of 1-Bromo-2-ethynylbenzene (4) (300 mg, 1.7 mmol) and iodoaryl compounds (1.1 mmol) in toluene (10 mL). The reaction mixture was stirred at 45 °C for 4 hours. After the mixture was cooled, water was added and the resulting mixture was extracted with methylene chloride. The combined organic layers were washed with water and brine, then dried over MgSO_4_, filtered, and concentrated in vacuo. The residue was purified by flash column chromatography using *n*-hexane as the eluent.

The 2-(2-Bromophenylethynyl)thiophene (2h). Yield: 93%; IR (neat, cm^−1^) 2205, 1419, 1214, 1026; ^1^H NMR (400 MHz, CDCl_3_) δ 7.58 (dd, *J* = 8.0, 1.0 Hz, 1H), 7.51 (dd, *J* = 7.7, 1.6 Hz, 1H), 7.31 (ddd, *J* = 6.2, 4.4, 1.0 Hz, 2H), 7.26 (td, *J* = 7.6, 1.2 Hz, 1H), 7.15 (td, *J* = 7.6, 1.6 Hz, 1H), 7.00 (dd, *J* = 5.2, 1.6 Hz, 1H); ^13^C NMR (100 MHz, CDCl_3_) δ 133.1, 132.5, 132.5, 129.5, 127.9, 127.2, 127.1, 125.3, 125.2, 122.8, 91.7, 87.2.

The 1-Bromo-2-(3-trifluoromethylphenylethynyl)benzene (2i). Yield: 89%; IR (neat, cm^−1^) 1713, 1338, 1128; ^1^H NMR (400 MHz, CDCl_3_) δ 7.82 (s, 1H), 7.73 (d, *J* = 7.6 Hz, 1H), 7.65–7.57 (m, 2H), 7.56 (dd, *J* = 7.6, 1.6 Hz, 1H), 7.47 (t, *J* = 7.7 Hz, 1H), 7.30 (td, *J* = 7.6, 1.2 Hz, 1H), 7.20 (td, *J* = 7.8, 1.6 Hz, 1H); ^13^C NMR (100 MHz, CDCl_3_) δ 134.8 (d, *J* = 4.8), 133.4, 132.5, 131.0 (q, *J* = 32.4), 129.9, 128.9, 128.4 (q, *J* = 3.8), 127.1, 125.7, 125.1 (q, *J* = 3.8), 124.8, 123.9, 123.7 (q, *J* = 271.0), 92.1, 89.4.

The 2-(2-Bromophenylethynyl)pyridine (2*J*). Yield: 94%; IR (neat, cm^−1^) 2920, 2224, 1578, 1472; ^1^H NMR (400 MHz, CDCl_3_) δ 8.63 (s, 1H), 7.69 (t, *J* = 7.6 Hz, 1H), 7.65–7.56 (m, 3H), 7.33-7.18 (m, 3H); ^13^C NMR (100 MHz, CDCl_3_) δ 150.1, 143.1, 136.2, 133.8, 132.5, 130.1, 127.5, 127.1, 125.9, 124.5, 123.0, 92.7, 87.6.

The 1-Bromo-2-(4-chlorophenylethynyl)benzene (2k). Yield: 90%; IR (neat, cm^−1^) 1486, 1085; ^1^H NMR (400 MHz, CDCl_3_) δ 7.61 (dd, *J* = 8.01, 0.8 Hz, 1H), 7.54 (dd, *J* = 7.6, 1.6 Hz, 1H), 7.5 (d, *J* = 8.6 Hz, 2H), 7.34 (d, *J* = 8.6 Hz, 2H), 7.29 (td, *J* = 7.6, 1.2 Hz, 1H), 7.19 (td, *J* = 7.86, 1.2 Hz, 1H); ^13^C NMR (100 MHz, CDCl_3_) δ 134.7, 133.2, 132.9, 132.5, 129.6, 128.7, 127.1, 125.6, 125.1, 121.4, 92.7, 88.9.

#### 3.1.5. General Procedure for the Preparation of Benzo[*b*]tellurophenes and Benzo[*b*]selenophenes (1a^−1^o)

A solution of NaHTe (0.6 mmol) which was freshly prepared from tellurium powder (47 mg) and NaBH_4_ (22 mg) in DMF (0.5 mL) was added to a solution of compound 2 (0.4 mmol) in DMF (4 mL) at 100 °C. The reaction mixture was stirred at 100 °C overnight. After addition of water, the aqueous mixture was extracted with methylene chloride. The organic layers were washed with water and brine, then dried over MgSO_4_, filtered, and concentrated in vacuo. The residue was purified by flash column chromatography using *n*-hexane or *n*-hexane /ethyl acetate as the eluent.

The 2-Phenylbenzo[*b*]tellurophene (1a) [27]. Yield: 50%; IR (neat, cm^−1^) 1482, 1428; ^1^H NMR (400 MHz, CDCl_3_) δ 7.99 (phenyl, s, 1H), 7.90 (ArH, d, *J* = 7.8 Hz, 1H), 7.80 (ArH, d, *J* = 7.8 Hz, 1H), 7.54 (ArH, d, *J* = 7.4 Hz, 2H), 7.40–7.28 (phenyl, m, 4H), 7.13 (ArH, t, *J* = 7.4 Hz, 1H); ^13^C NMR (100 MHz, CDCl_3_) δ 149.1, 142.5, 139.7, 132.2, 131.9, 131.8, 129.0, 128.1, 127.7, 127.5, 125.6, 124.5.

Benzo[*b*]tellurophen-2-ylmethanol (1b). Yield: 50%; IR (neat, cm^−1^) 3224, 2918, 2848, 1444; ^1^H NMR (400 MHz, CDCl_3_) δ 7.89 (ArH, d, *J* = 7.6 Hz, 1H), 7.70 (ArH, d, *J* = 7.6 Hz, 1H), 7.63 (tellurophene, s, 1H), 7.34 (ArH, td, *J* = 7.6, 1.2, 1H), 7.10 (ArH, td, *J* = 7.6, 1.2, 1H), 4.87 (CH_2_, d, *J* = 0.8 Hz, 2H), 2.37 (s, br OH); ^13^C NMR (100 MHz, CDCl_3_) δ 148.0, 146.2, 132.1, 132.0, 131.3, 127.5, 125.3, 124.1, 65.9; HRMS(ESI): m/z 261.9622 [M+H]^+^ (calcd for C9H9OTe^+^ 261.9637).

The tert-Butyl benzo[*b*]tellurophen-2-ylmethylcarbamate (1c). Yield: 56%; IR (neat, cm^−1^) 3298, 2970, 1661, 1523, 1288; ^1^H NMR (400 MHz, CDCl_3_) δ 7.83 (ArH, d, *J* = 7.8 Hz, 1H), 7.66 (ArH, d, *J* = 7.8 Hz, 1H), 7.55 (tellurophene, s, 1H), 7.31 (ArH, t, *J* = 7.6 Hz, 1H), 7.06 (ArH, t, *J* = 7.6 Hz, 1H), 5.20 (s, NH), 4.43 (CH_2_, d, *J* = 6.0 Hz, 2H), 1.48 (3XCH_3_, s, 9H); ^13^C NMR (100 MHz, CDCl_3_) δ 156.1, 147.2, 141.9, 133.6, 133.3, 131.9, 127.3, 125.2, 124.3, 80.1, 46.4, 28.5; HRMS(ESI): m/z 384.0217 [M+Na]^+^ (calcd for C14H18NNaO_2_Te+ 384.0214).

Methyl benzo[*b*]tellurophen-2-ylmethylcarbamate (1d). Yield: 62%; IR (neat, cm^−1^) 3334, 2951, 1667, 1521, 1260; ^1^H NMR (400 MHz, CDCl_3_) δ ^1^H NMR (400 MHz, CDCl_3_) δ 7.84 (ArH, d, *J* = 7.8 Hz, 1H), 7.67 (ArH, d, *J* = 7.8 Hz, 1H), 7.59 (tellurophene, s, 1H), 7.32 (ArH, t, *J* = 7.6 Hz, 1H), 7.09 (ArH, t, *J* = 7.6 Hz, 1H), 5.33 (s, NH), 4.52 (CH_2_, d, *J* = 5.6 Hz, 2H), 3.71 (CH_3_, s, 3H); ^13^C NMR (100 MHz, CDCl_3_) δ 157.0, 147.3, 141.1, 133.9, 133.0, 132.0, 127.4, 125.3, 124.4, 52.5, 46.7; HRMS(ESI): m/z 341.9744 [M+Na]^+^ (calcd for C_14_H_11_NNaO_2_Te^+^ 342.9742).

Ethyl benzo[*b*]tellurophen-2-ylmethylcarbamate (1e). Yield: 49%; IR (neat, cm^−1^) 3318, 2914, 1673, 1522, 1253; ^1^H NMR (400 MHz, CDCl_3_) δ 7.84 (ArH, d, *J* = 7.8 Hz, 1H), 7.68 (ArH, d, *J* = 7.8 Hz, 1H), 7.59 (tellurophene, s, 1H), 7.32 (ArH, t, *J* = 7.6 Hz, 1H), 7.09 (ArH, t, *J* = 7.6 Hz, 1H), 5.30 (s, 1H), 4.51 (CH_2_, d, *J* = 6.2 Hz, 2H), 4.17 (CH_2_, q, *J* = 7.0 Hz, 2H), 1.26 (CH_2_, t, *J* = 7.0 Hz, 3H); ^13^C NMR (100 MHz, CDCl_3_) δ 156.7, 147.2, 141.3, 133.8, 133.1, 132.0, 127.3, 125.3, 124.4, 61.3, 46.6, 14.7.; HRMS(ESI): m/z 355.9916 [M+Na]^+^ (calcd for C_12_H_13_NNaO_2_Te^+^ 355.9906).

Isobutyl benzo[*b*]tellurophen-2-ylmethylcarbamate (1f). Yield: 64%; IR (neat, cm^−1^) 3364, 1678, 1513, 1246, 1163; ^1^H NMR (400 MHz, CDCl_3_) δ 7.84 (ArH, d, *J* = 7.8 Hz, 1H), 7.68 (ArH, d, *J* = 7.8 Hz, 1H), 7.60 (tellurophene, s, 1H), 7.32 (ArH, t, *J* = 7.6 Hz, 1H), 7.09 (ArH, t, *J* = 7.6 Hz, 1H), 5.31 (NH, 1H), 4.52 (CH_2_, d, *J* = 6.2 Hz, 2H), 3.90 (CH_2_, d, *J* = 6.6 Hz, 2H), 1.93 (CH, sep, 6.6 Hz, 1H), 0.92 (2XCH_3_, d, *J* = 6.6 Hz, 6H); ^13^C NMR (100 MHz, CDCl_3_) δ 156.9, 147.2, 141.3, 133.8, 133.1, 132.0, 127.3, 125.3, 124.4, 71.5, 46.6, 28.0, 19.1; HRMS(ESI): m/z 384.0208 [M+Na]+ (calcd for C_14_H_17_NNaO_2_Te^+^ 384.0214).

Neopentyl benzo[*b*]tellurophen-2-ylmethylcarbamate (1g). Yield: 71%; IR (neat, cm^−1^) 3296, 2955, 1667, 1533; ^1^H NMR (400 MHz, CDCl_3_) δ 7.86 (ArH, d, *J* = 7.8 Hz, 1H), 7.70 (ArH, d, *J* = 7.8 Hz, 1H), 7.63 (tellurophene, s, 1H), 7.33 (ArH, t, *J* = 7.6 Hz, 1H), 7.10 (ArH, t, *J* = 7.6 Hz, 1H), 5.27 (NH, 1H), 4.56 (CH_2_, d, *J* = 6.2 Hz, 2H), 3.84 (CH_2_, s, 2H), 0.94 (2XCH_3_, s, 9H); ^13^C NMR (100 MHz, CDCl_3_) δ 156.9, 147.2, 141.2, 133.8, 133.1, 131.9, 127.3, 125.2, 124.4, 74.6, 46.6, 31.5, 26.4; HRMS(ESI): m/z 378.0372 [M+Na]^+^ (calcd for C_15_H_19_NNaO_2_Te+ 378.0376).

The 2-Benzo[*b*]tellurophen-2-ylthiophene (1h). Yield: 54%; IR (neat, cm^−1^)1580,1497; ^1^H NMR (400 MHz, CDCl_3_) δ 7.86–7.81 (m, 2H), 7.73 (ArH, d, *J* = 7.8 Hz, 1H), 7.34 (ArH, t, *J* = 7.5 Hz, 1H), 7.26–7.23 (thiophene, m, 1H), 7.11 (ArH, t, *J* = 7.6 Hz, 1H), 7.05(thiophene, d, *J* = 2.8 Hz, 1H), 7.00 (thiophene, t, *J* = 3.9 Hz, 1H); ^13^C NMR (100 MHz, CDCl3) δ 148.5, 143.6, 132.0, 131.8, 131.8, 131.7, 127.8, 127.5, 126.5, 125.7, 125.4, 124.6; HRMS(ESI): m/z 311.9402 [M+H]^+^ (calcd for C_12_H_8_STe^+^ 313.9409).

The 2,3-Trifluoromethylphenylbenzo[*b*]tellurophene (1i). Yield: 40%; IR (neat, cm^−1^) 132, 1171, 1110; ^1^H NMR (400 MHz, CDCl_3_) δ 8.02 (phenyl, s, 1H), 7.90 (ArH, d, *J* = 7.8 Hz, 1H), 7.82 (ArH, d, *J* = 7.8 Hz, 1H), 7.77 (tellurophene, s, 1H), 7.65 (phenyl, d, *J* = 7.6 Hz, 1H), 7.56 (phenyl, d, *J* = 7.6 Hz, 1H), 7.48 (phenyl, t, *J* = 7.6 Hz, 1H), 7.40 (t, *J* = 7.6 Hz, 1H), 7.16 (t, *J* = 7.6 Hz, 1H); ^13^C NMR (100 MHz, CDCl_3_) δ 148.8, 141.0, 140.1, 133.2, 132.7, 131.9, 131.4 (q, *J* = 31.2 Hz), 130.9, 129.5, 128.1, 125.8, 125.0, 124.5 (q, *J* = 3.8 Hz), 123.9 (q, *J* = 271.0Hz), 123.7 (q, *J* = 3.8 Hz); HRMS(ESI): m/z 375.9791 [M+H]^+^ (calcd for C_15_H_10_F_3_Te^+^ 376.9797).

The 2-Benzo[*b*]tellurophen-2-ylpyridine (1*J*). Yield: 40%; IR (neat, cm^−1^) 2919, 1580, 1455, 1426; ^1^H NMR (400 MHz, CDCl_3_) δ 8.48 (pyridine, d, *J* = 2.4 Hz 1H), 8.30 (tellurophene, s, 1H), 7.92 (ArH, d, *J* = 7.8 Hz, 1H), 7.84–7.77 (m, 2H), 7.67 (pyridine, t, *J* = 7.6 Hz, 1H), 7.35 (ArH, t, *J* = 7.6 Hz, 1H), 7.19–7.10 (m, 2H); ^13^C NMR (100 MHz, CDCl_3_) δ 156.4, 149.8, 146.6, 136.3, 133.5, 132.2, 131.6, 128.3, 125.3, 124.9, 122.6, 116.9; HRMS(ESI): m/z 309.9870 [M+H]^+^ (calcd for C_13_H_10_NTe^+^ 309.9875).

The 2-(4-Chlorophenyl)benzo[*b*]tellurophene (1k) [27]. Yield: 41%; IR (neat, cm^−1^) 1091; ^1^H NMR (400 MHz, CDCl_3_) δ 7.93 (tellurophene, s, 1H), 7.87 (ArH, d, *J* = 7.8 Hz, 1H), 7.78 (ArH, d, *J* = 7.8 Hz, 1H), 7.46–7.40 (m, 2H), 7.40–7.29 (m, 3H), 7.12 (ArH, td, *J* = 7.9, 1.2 Hz, 1H); ^13^C NMR (100 MHz, CDCl_3_) δ 149.0, 140.7, 138.3, 133.9, 132.4, 132.4, 131.9, 129.0, 128.6, 127.86, 125.7, 124.7; HRMS(ESI): m/z 341.9529 [M+H]^+^ (calcd for C_14_H_10_ClTe^+^ 342.9533).

The 2-Phenylbenzo[*b*]selenophene (1l). Yield: 63%; IR (neat, cm^−1^) 1442, 926; ^1^H NMR (400 MHz, CDCl_3_) δ 7.85 (ArH, d, *J* = 8.0 Hz, 1H), 7.76 (ArH, d, *J* = 7.8 Hz, 1H), 7.69 (selenophene, s, 1H), 7.63 (ArH, d, *J* = 7.6 Hz, 2H), 7.43–7.35 (m, 2H), 7.36–7.28 (m, 2H), 7.23 (ArH, t, *J* = 7.4 Hz, 1H); ^13^C NMR (100 MHz, CDCl_3_) δ 147.7, 143.3, 140.9, 136.2, 128.9, 128.3, 126.9, 125.4, 125.4, 124.8, 124.5, 123.1; HRMS(ESI): m/z 259.0020 [M+H]+ (calcd for C_14_H_11_Se^+^ 259.0026).

The tert-Butyl benzo[*b*]selenophen-2-ylmethylcarbamate (1m). Yield: 59%; IR (neat, cm^−1^) 3416, 2976, 1681, 1499, 1158; ^1^H NMR (400 MHz, CDCl_3_) δ 7.83 (ArH, d, *J* = 8.0 Hz, 1H), 7.68 (ArH, d, *J* = 7.8 Hz, 1H), 7.37–7.23 (m, 2H), 7.22 (ArH, t, *J* = 7.6 Hz, 1H), 5.01 (s, NH), 4.56 (CH_2_, d, *J* = 5.2 Hz, 2H), 1.48 (3XCH_3_, s, 9H); ^13^C NMR (100 MHz, CDCl_3_) δ 155.6, 147.1, 141.8, 141.4, 125.5, 125.2, 125.0, 124.6, 124.4, 79.9, 42.6, 28.4; HRMS(ESI): m/z 334.0312 [M+Na]^+^ (calcd for C_14_H_17_NNaO_2_Se^+^ 334.0317).

The 2-Benzo[*b*]selenophen-2-ylpyridine (1n). Yield: 23%; IR (neat, cm^−1^) 2919, 1457, 1426; ^1^H NMR (400 MHz, CDCl_3_) δ 8.58 (pyridine, d, *J* = 4.8 Hz, 1H), 7.97 (s, 1H), 7.90 (ArH, d, *J* = 8.0 Hz, 1H), 7.82–7.76 (m, 2H), 7.69 (ArH, td, *J* = 7.6, 1.6 Hz, 1H), 7.29–7.24 (m, 1H), 7.17 (ArH, ddd, *J* = 7.2, 5.2, 0.8, 1H); ^13^C NMR (100 MHz, CDCl_3_) δ 153.9, 149.7, 149.0, 143.3, 142.0, 136.4, 125.9, 125.7, 125.1, 124.7, 124.1, 122.6, 118.6; HRMS(ESI): m/z 259.9969 [M+H]^+^ (calcd for C_13_H_9_NSe^+^ 259.9973).

The 2-(4-Chlorophenyl)benzo[*b*]selenophene (1o). Yield: 70%; IR (neat, cm^−1^) 2918, 1091; ^1^H NMR (400 MHz, CDCl_3_) δ 7.86 (ArH, d, *J* = 8.0 Hz, 1H), 7.76 (ArH, d, *J* = 8.0 Hz, 1H), 7.67 (selenophene, s, 1H), 7.55 (phenyl, d, *J* = 8.6 Hz, 2H), 7.39–7.33 (m, 3H), 7.29–7.22 (m, 1H); ^13^C NMR (100 MHz, CDCl_3_) δ 146.1, 143.1, 141.0, 134.7, 134.1, 129.1, 128.0, 125.5, 125.4, 125.0, 124.7, 123.5; HRMS(ESI): m/z 291.9632 [M+H]^+^ (calcd for C_14_H_10_ClSe^+^ 292.9636).

#### 3.1.6. Procedure for the Preparation of Benzo[*b*]furans (1p, 1q)

The 2-Phenylbenzofuran (1p). Ethynylbenzene (366 mg, 3.5 mmol), PdCl_2_(Ph_3_P)_2_ (123 mg, 0.2 mmol), and CuI (34 mg, 0.2 mmol) were added to a solution of 2-iodophenol (773 mg, 3.5 mmol) in toluene (4 mL). The reaction mixture was stirred at 45 °C for 4 hours. After the mixture was cooled, water was added and the resulting mixture was extracted with methylene chloride. The combined organic layers were washed with water and brine, then dried over MgSO_4_, filtered, and concentrated in vacuo. The residue was purified by flash column chromatography using *n*-hexane as the eluent. Yield: 85%; IR (neat, cm^−1^) 1603, 1452; ^1^H NMR (400 MHz, CDCl_3_) δ 7.86 (ArH, d, *J* = 8.0 Hz, 2H), 7.58 (ArH, d, *J* = 7.6 Hz, 1H), 7.52 (ArH, d, *J* = 8.0 Hz, 1H), 7.44 (ArH, t, *J* = 7.6 Hz, 2H), 7.35 (ArH, t, *J* = 7.4 Hz, 1H), 7.30-7.19 (m, 2H), 7.02 (furan, s, 1H);^13^C NMR (100 MHz, CDCl_3_) δ 155.9, 154.9, 130.5, 129.2, 128.8, 128.5, 124.9, 124.2, 122.9, 120.9, 111.2, 101.3.

The tert-Butyl benzofuran-2-ylmethylcarbamate (1q). Di-tert-butyl dicarbonate (362 mg, 1.66 mmol) was slowly added to a solution of propagyl amine (257 mg, 1.66 mmol) in piperidine (4 mL) at 0 °C. After the reaction mixture was stirred at room temperature for 3 hours, water was added to the mixture, and the resulting mixture was extracted with methylene chloride. The combined organic layers were washed with water and brine, then dried over MgSO_4_, filtered, and concentrated in vacuo. Without purification, the resulting mixture was dissolved with toluene (10 mL) and mixed with piperidine (2 mL) 2-iodophenol (365 mg, 1.66 mmol), PdCl_2_(Ph_3_P)_2_ (58 mg, 0.08 mmol), and CuI (15 mg, 0.08 mmol) Then the reaction mixture was stirred at 45 °C for 4 hours. After the mixture was cooled, water was added and the resulting mixture was extracted with methylene chloride. The combined organic layers were washed with water and brine, then dried over MgSO_4_, filtered, and concentrated in vacuo. The residue was purified by flash column chromatography using *n*-hexane: ethyl acetate = 6:1 as the eluent. Yield: 90%; IR (neat, cm^−1^) 3371, 2984, 1681, 1508, 1453; ^1^H NMR (400 MHz, CDCl_3_) δ 7.51 (ArH, d, *J* = 7.6 Hz, 1H), 7.43 (ArH, d, *J* = 7.6 Hz, 1H), 7.25 (ArH, td, *J* = 7.3, 1.3 Hz, 1H), 7.20 (ArH, td, *J* = 7.3, 0.8 Hz, 1H), 6.57 (furan, s, 1H), 5.20 (s, NH), 4.44 (CH_2_, d, *J* = 5.6 Hz, 2H), 1.47 (3XCH_3_, s, 9H); ^13^C NMR (100 MHz, CDCl_3_) δ 155.7, 154.9, 154.9, 128.3, 124.0, 122.7, 120.9, 111.1, 103.6, 79.8, 38.2, 28.4; HRMS(ESI): m/z 247.1260 [M]^+^ (calcd for C_14_H_17_NO_3_^+^ 247.1208).

### 3.2. Biology

#### 3.2.1. Acid Extraction of Histones

Acid extraction of histones was performed as described previously [28]. Briefly, HeLa cell pellets were suspended in 10 volumes of PBS and centrifuged at 200 × g for 10 min. Cells were then suspended with five volumes of hypotonic lysis buffer (10 mM Tris-Cl (pH 8.0), 1.5 mM MgCl_2_, 1 mM KCl, 1 mM DTT, and 1 mM phenylmethylsulfonyl fluoride) and 0.4 N H_2_SO_4_ at a final concentration of 0.2 M and subsequently lysed on ice for 30 min. After centrifugation at 16,000× *g* for 10 min at 4 °C, the cell supernatant fraction that contained acid-soluble proteins was retained. Trichloroacetic acid was added to the supernatant up to 33% and the samples were incubated on ice overnight. Proteins were pelleted by centrifugation at 16,000× *g* for 10 min at 4 °C and washed 4 times with ice cold acetone with centrifugations at 16,000× *g* for 5 min at 4 °C. Each histone pellet was air-dried for 20 min at room temperature and dissolved in an appropriate volume of ddH_2_O.

#### 3.2.2. Western Blot Analysis

Acid extracted proteins were analyzed by sodium dodecyl sulfate-polyacrylamide gel electrophoresis (SDS-PAGE)/immunoblotting with antibodies recognizing methylated histones H3K9 and histone H3. Protein samples were separated along with molecular weight markers (Bio-Rad, Hercules, CA, USA) in 15% polyacrylamide gels. Gels were transferred onto 0.45-μm nitrocellulose membranes (Schleicher and Schuell). Species-specific immunoglobulin G-horseradish peroxidase (IgG-HRP) secondary antibodies were purchased from Bio-Rad. Blots were developed with chemiluminescent substrate (Amersham International, Buckinghamshire, England), and autoradiography was performed utilizing X-OMAT film (Kodak, Rochester, NY, USA). The blots were semi‑quantified using FusionCapt software version 16.08a (Viber Lourmat Sté, Collégien, France).

#### 3.2.3. Histone Demethylase Assay

Histone demethylase activity assay was performed according to the manufacturer’s instruction (Cayman’s demethylase (*J*umon*J*i-type) activity assay kit, 700390) and used to determine demethylase activity and to select potent inhibitors. All compounds were dissolved in dimethyl sulfoxide (DMSO) at various concentrations and added to the mixture such that the final DMSO concentration was 1%. All solutions were kept on ice until being mixed and incubated at 37 °C. Recombinant enzyme KDM4A, cofactor mixture (2-oxoglutarate, ascorbate, Fe(II)), H3K9me3 peptide as substrate, and compound were incubated for 60 min at 37 °C; the final volume was 50 μL. Then, 40 μL of ammonium acetate and 10 μL of detector were added to all of the wells and incubated for 15 min at 25 °C. The resulting fluorescent production was analyzed using an excitation wavelength 370 and an emission wavelength 470 nM. All data were processed with GraphPad Prism 5.0™.

#### 3.2.4. MTT Assay

Cell viability was assessed by 3-(4,5-dimethylthiazol-2-yl)-2, 5-diphenyltetrazo-lium bromide (MTT) assay. Briefly, 8 × 10^4^ of cells were seeded into 96-well plates for 24 h, followed by incubation with a compound for 72 h. After adding 20 µL/well of MTT solution, the cells were incubated for another 2 h. Supernatants were then removed and the formazan crystals were dissolved in 100 µL/well DMSO. The absorbance at 570/630 nm of each sample was measured using multimode microplate reader (Teckan, Switzerland). Three independent triplicate experiments were performed.

#### 3.2.5. Statistical Analysis

Statistical analyses were performed using GraphPad Prism software (GraphPad Software 5.0, Inc., La Jolla, CA, USA). All data are presented as the mean ± SD from three independent experiments. Statistical significances were determined by one-way analysis of variance with post hoc analysis using Tukey’s multiple comparison test. *p* < 0.05 was considered to indicate a statistically significant difference.

## 4. Conclusions

In conclusion, benzo[*b*]tellurophene, benzo[*b*]selenophene, and benzo[*b*]furan compounds were efficiently synthesized and evaluated for their histone demethylase inhibition activity. In particular, carbamate derivatives of benzo[*b*]tellurophene are newly synthesized novel compounds, and novel carbamate derivative compound 1c exhibited a specific KDM4 inhibitory activity, without affecting the activity of other histone modifying enzymes. We also confirmed that compound 1c induced selective cell death in cancer cells, but it is not cytotoxic to normal human cells. Compound 1c is therefore valuable for further biological investigation, particularly with regard to KDM4 inhibition, and it has potential to be an anticancer agent. This is the first report on the biological activity of benzo[*b*]tellurophene derivatives and the study of the mechanism of benzo[*b*]tellurophene inhibition of KDM4 is ongoing. The newly synthesized benzo[*b*]tellurophene or benzo[*b*]selenophene can be provided as a novel molecular framework that is similar to but distinct from benzofuran or benzothiophene.

## Figures and Tables

**Table 1:** Synthesized benzo[*b*]tellurophen, benzo[*b*]selenophen, and benzo[*b*]furan derivatives and their inhibitory activities on histone H3 lysine 9 demethylase (KDM4).

Cpds	Structure	Activity *	Cpds	Structure	Activity *
**1a**	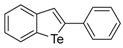	1.03	**1j**	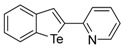	1.06
**1b**	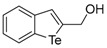	1.00	**1k**	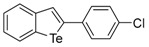	0.98
**1c**	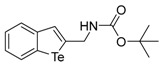	1.79	**1l**	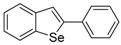	0.97
**1d**	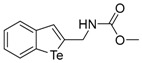	1.06	**1m**	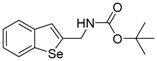	0.90
**1e**	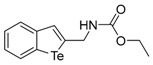	1.03	**1n**	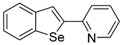	1.04
**1f**	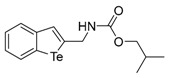	1.30	**1o**	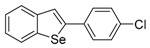	0.86
**1g**	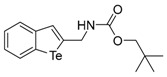	0.88	**1p**	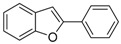	0.96
**1h**	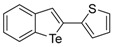	1.29	**1q**	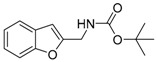	1.13
**1i**	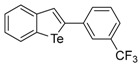	1.63			

* Activity is noted as the relative H3K9me3 level normalized to histone H3 levels compared to NC group.

## Supplementary Materials

Supplementary materials can be found at https://www.mdpi.com/1422-0067/20/23/5908/s1.
