# Supplementary material for: Benzo[b]tellurophenes as a Potential Histone H3 Lysine 9 Demethylase (KDM4) Inhibitor
Source: Int J Mol Sci. 2019 Nov 25;20(23):5908. doi: 10.3390/ijms20235908 (PMC6928947; doi:10.3390/ijms20235908)

# Supplementary data

*Benzo[b]tellurophene and benzo[b]selenophene compounds as a potential histone H3 Lysine 9 demethylase (KDM4) inhibitor*

**Yoon-Jung Kim [1], Dong Hoon Lee [1], Yong-Sung Choi [1], So Hee Kwon [1,2]* and Jin-Hyun Jeong [1,*]**

[1] College of Pharmacy, Yonsei Institute of Pharmaceutical Sciences, Yonsei University, 85 Songdogwahak-ro, Yeonsu-gu, Incheon 21983, Republic of Korea; sunshine333@sookmyung.ac.kr (Y.-J.K.); uriys2@gmail.com (Y.-S.C.); tci30@naver.com (D.-H.L)

[2] Department of Integrated OMICS for Biomedical Science, Yonsei University, Seoul 03720, Republic of Korea

* Correspondence: soheekwon@yonsei.ac.kr; Tel.: +82-32-749-4513 (S.-H.K); organicjeong@yonsei.ac.kr; Tel.: +82-32-749-4509 (J.-H.J)

* Corresponding author:

Professor So Hee Kwon
College of Pharmacy, Yonsei Institute of Pharmaceutical Sciences,Yonsei University,
85 Songdogwahak-ro, Yeonsu-gu, Incheon 406-840, Republic of Korea
Department of Integrated OMICS for Biomedical Science, Yonsei University, Seoul 03720, Republic of Korea

Tel.: +82-32-749-4513

e-mail: soheekwon@yonsei.ac.kr

Professor Jin-Hyun Jeong
College of Pharmacy, Yonsei Institute of Pharmaceutical Sciences,Yonsei University,
85 Songdogwahak-ro, Yeonsu-gu, Incheon 406-840, Republic of Korea

Tel.: +82-32-749-4509

e-mail: organicjeong@yonsei.ac.kr

$^{1}$H NMR of **1a**

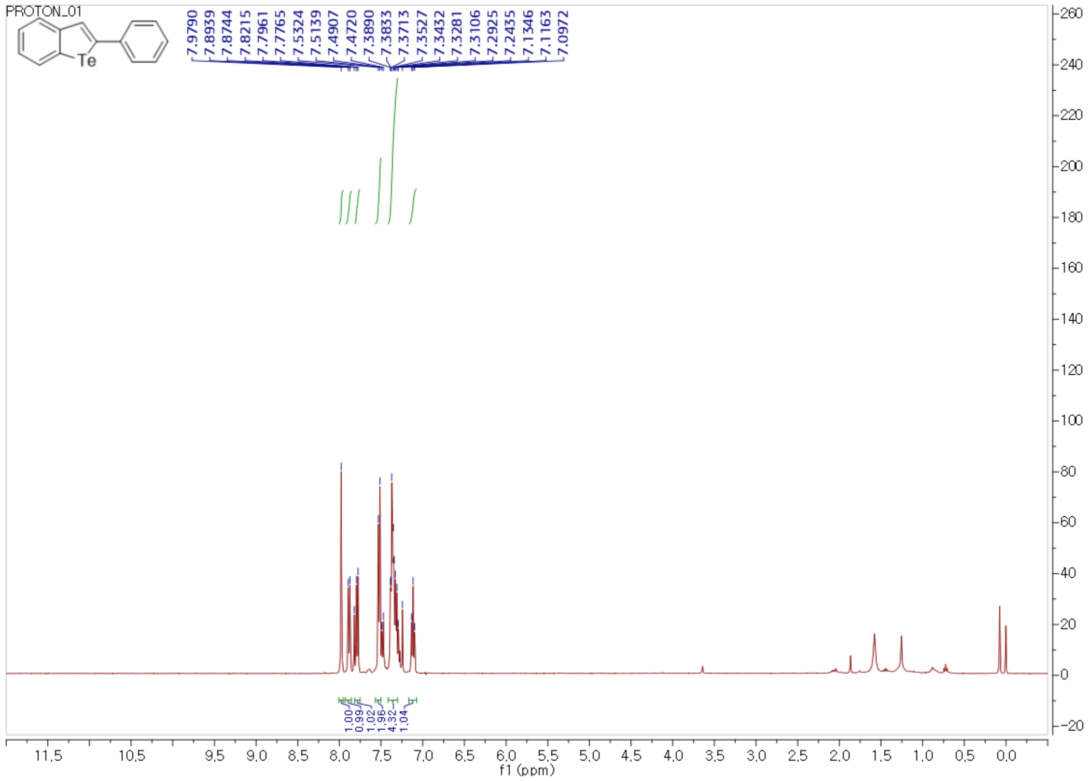


$^{13}$C NMR of **1a**

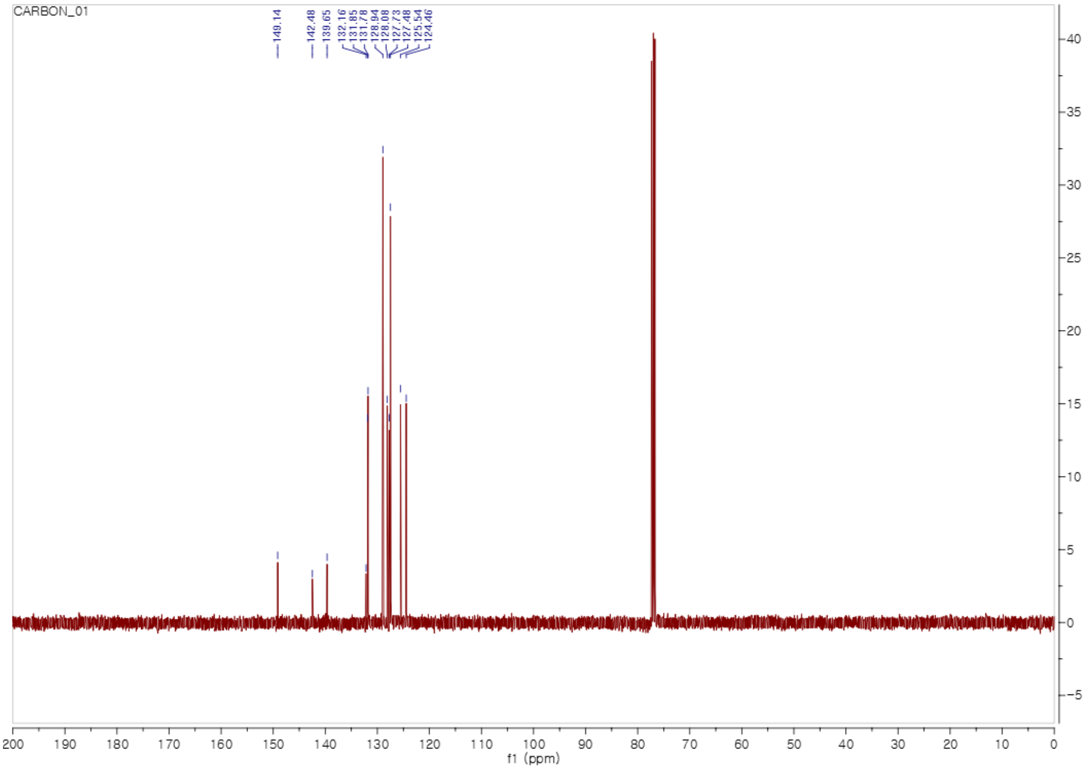

$^{1}$H NMR of **1b**

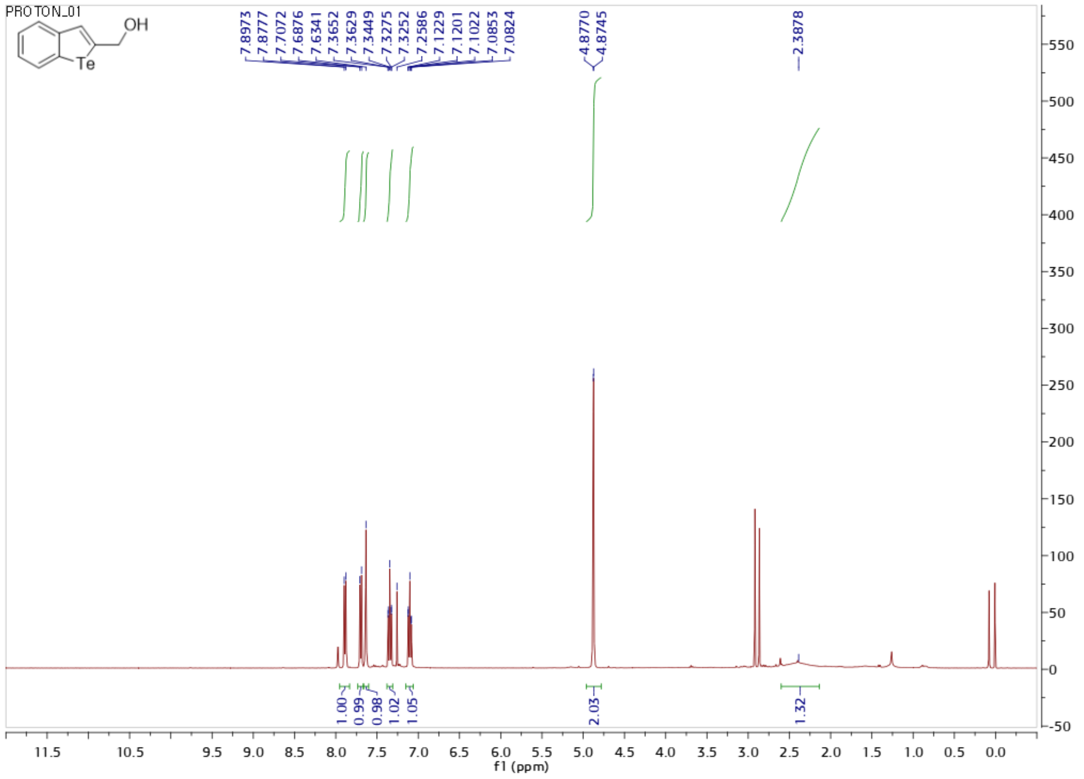


$^{13}$C NMR of **1b**

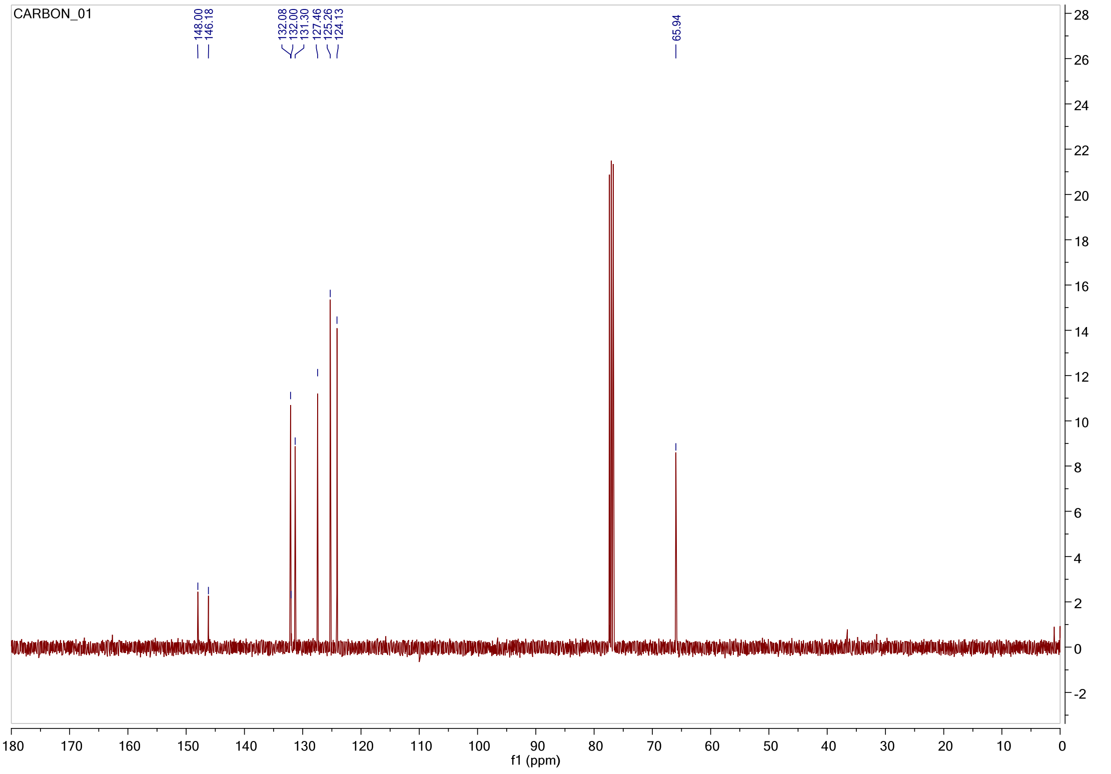

$^{1}$H NMR of **1c**

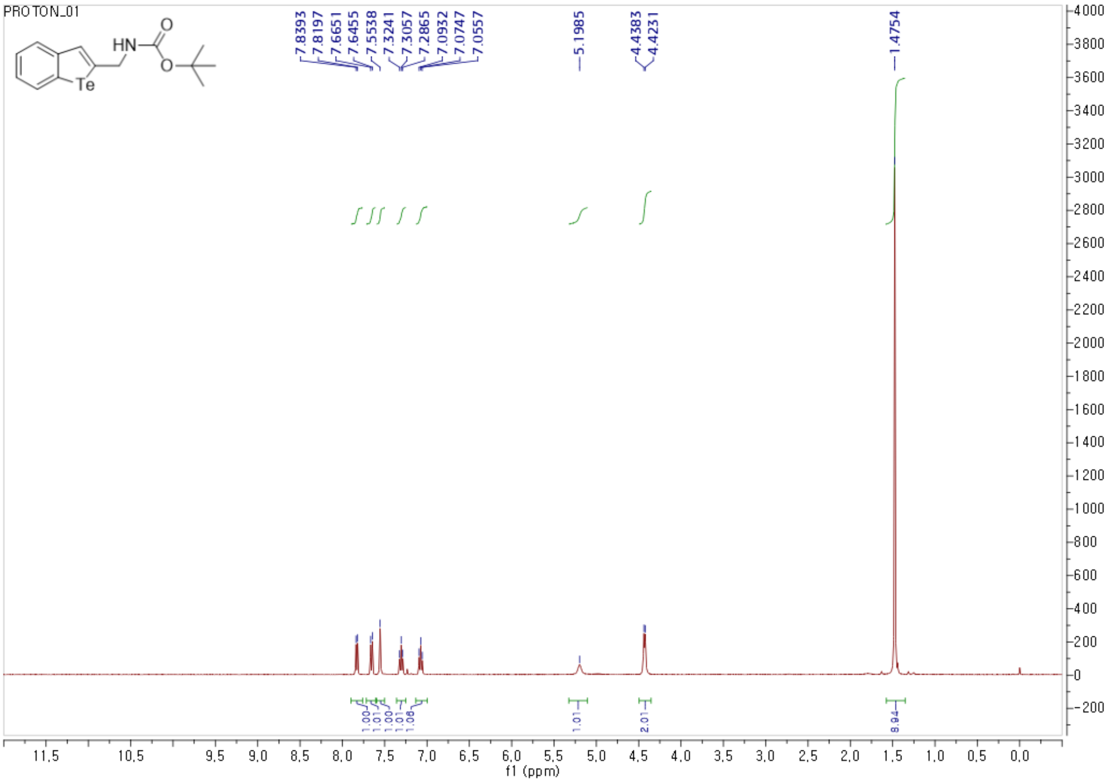


$^{13}$C NMR of **1c**

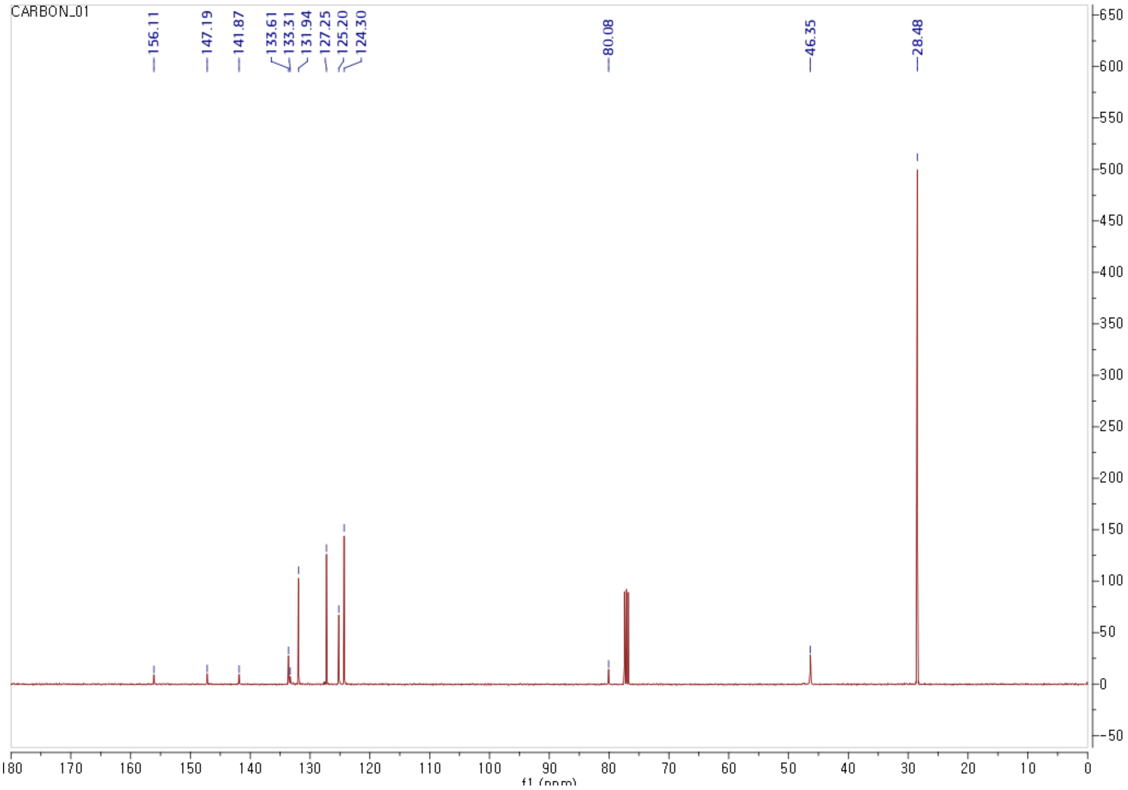

$^{1}$H NMR of **1d**

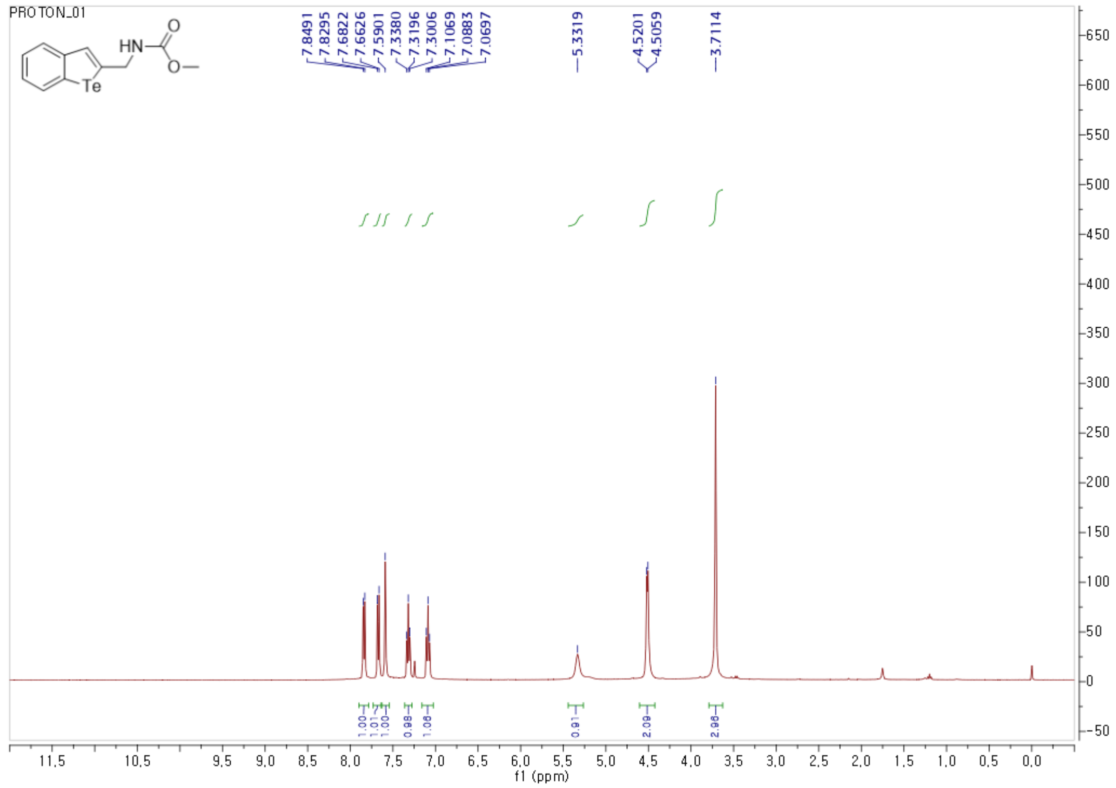


$^{13}$C NMR of **1d**

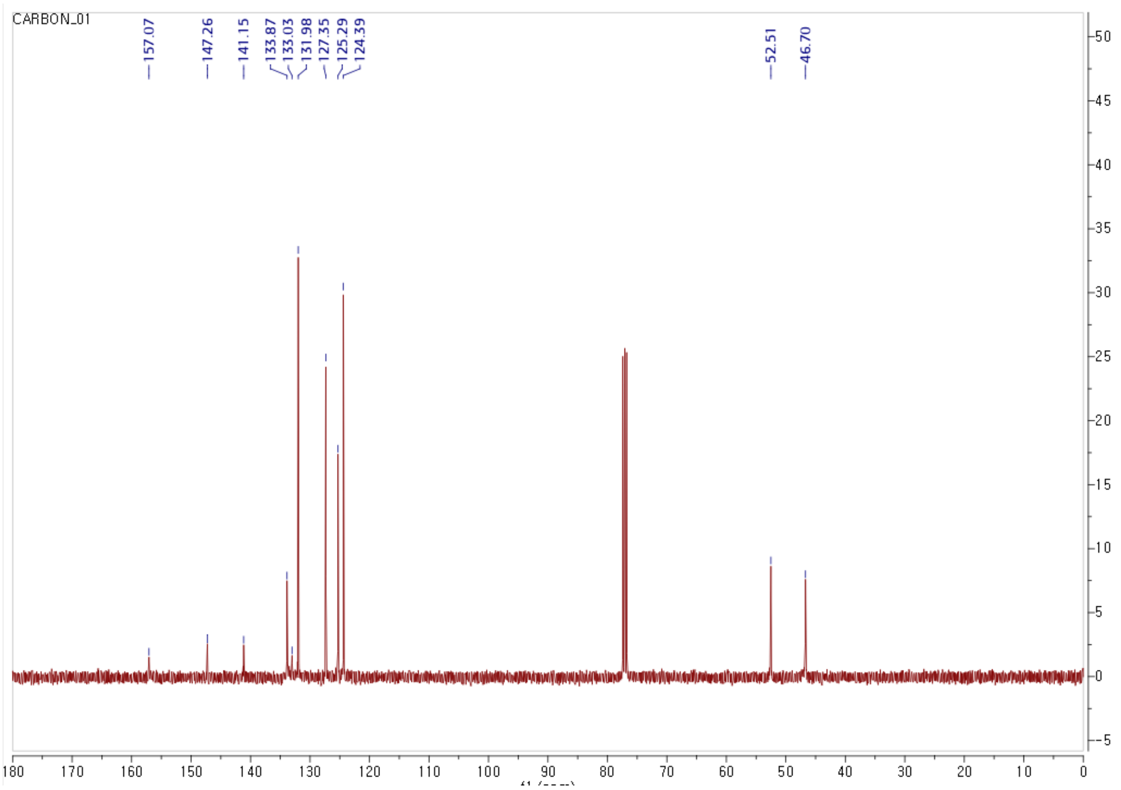

$^{1}$H NMR of **1e**

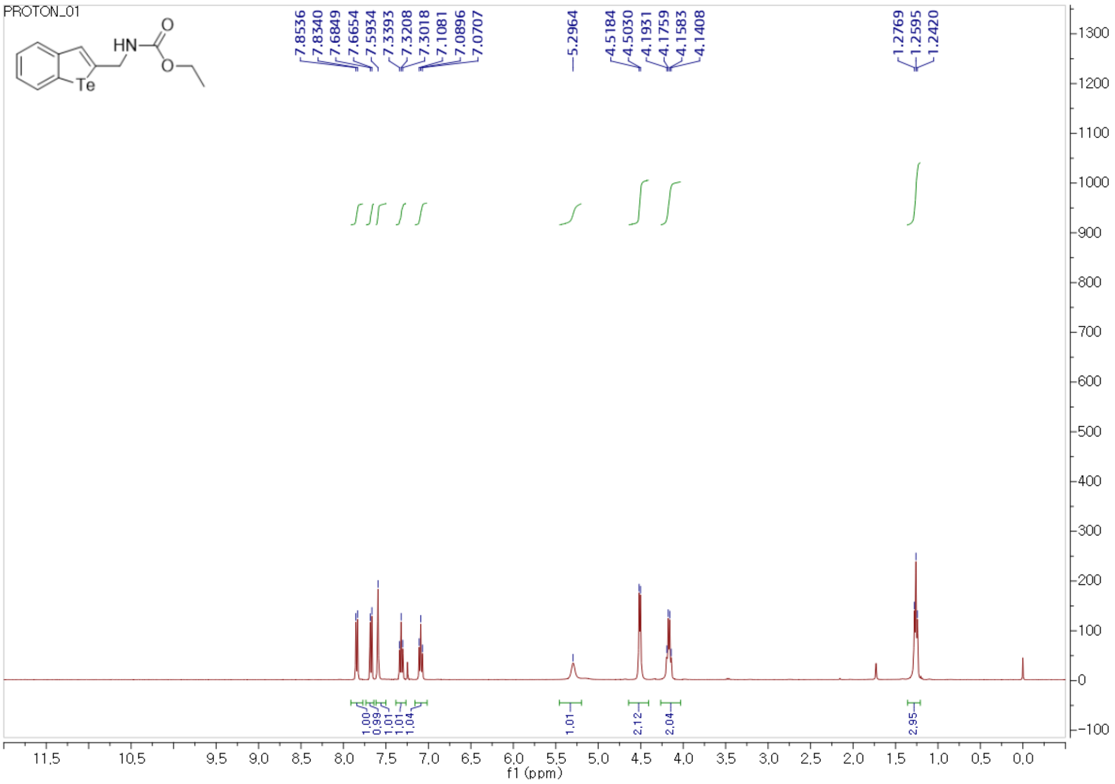


$^{13}$C NMR of **1e**

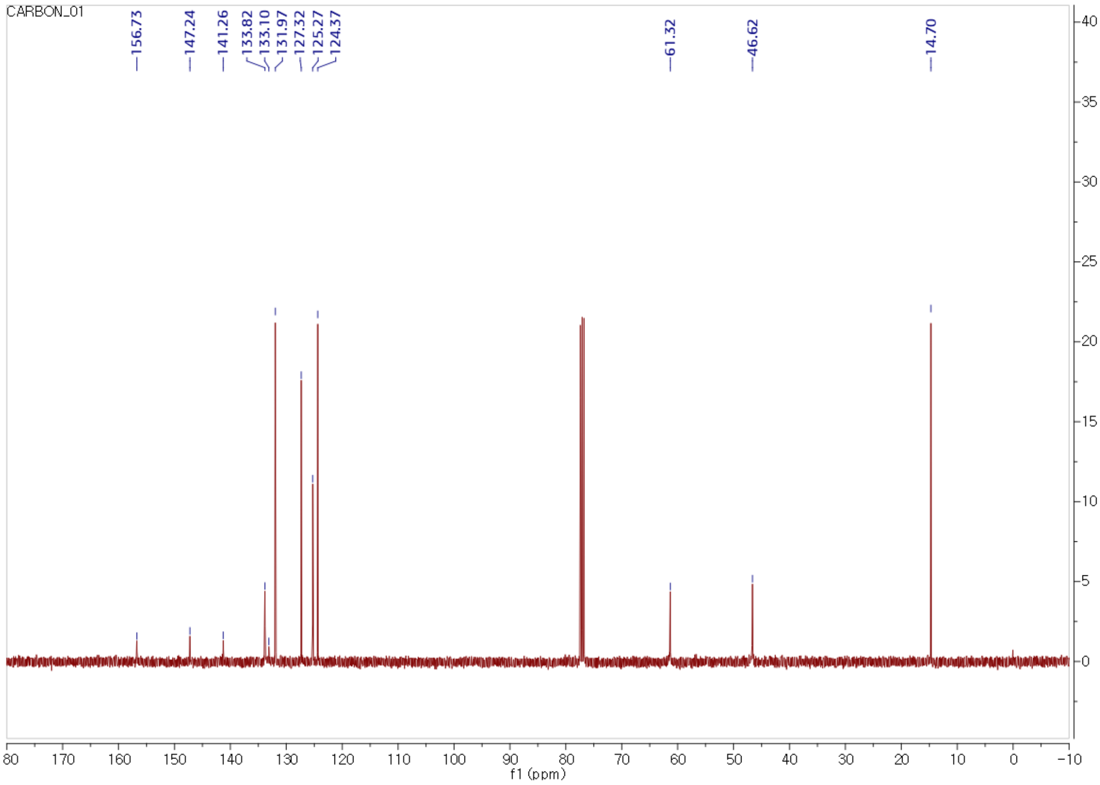

$^{1}$H NMR of **1f**

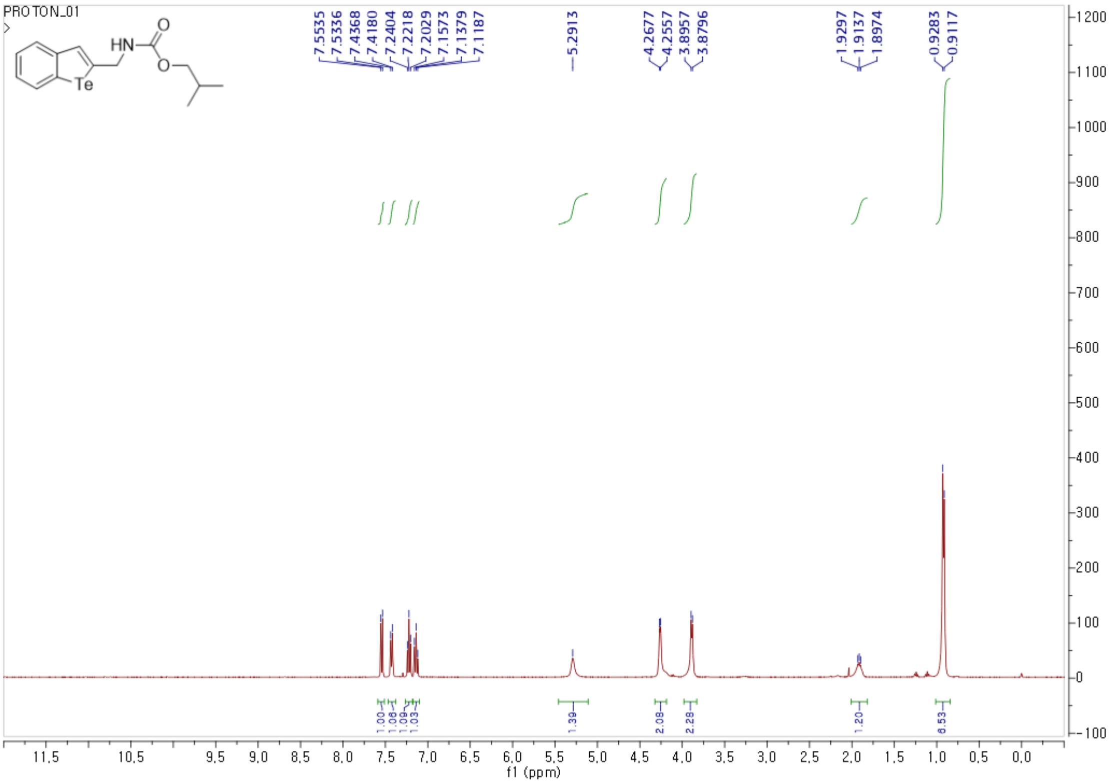


$^{13}$C NMR of **1f**

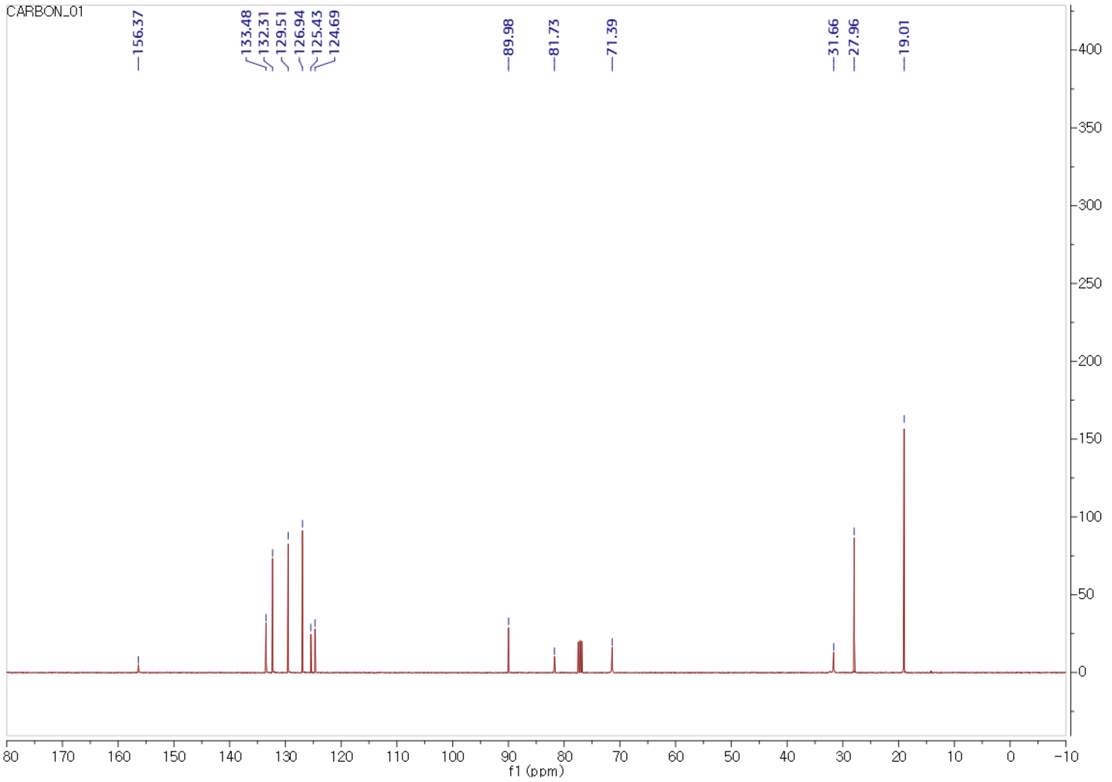

$^{1}$H NMR of **1g**

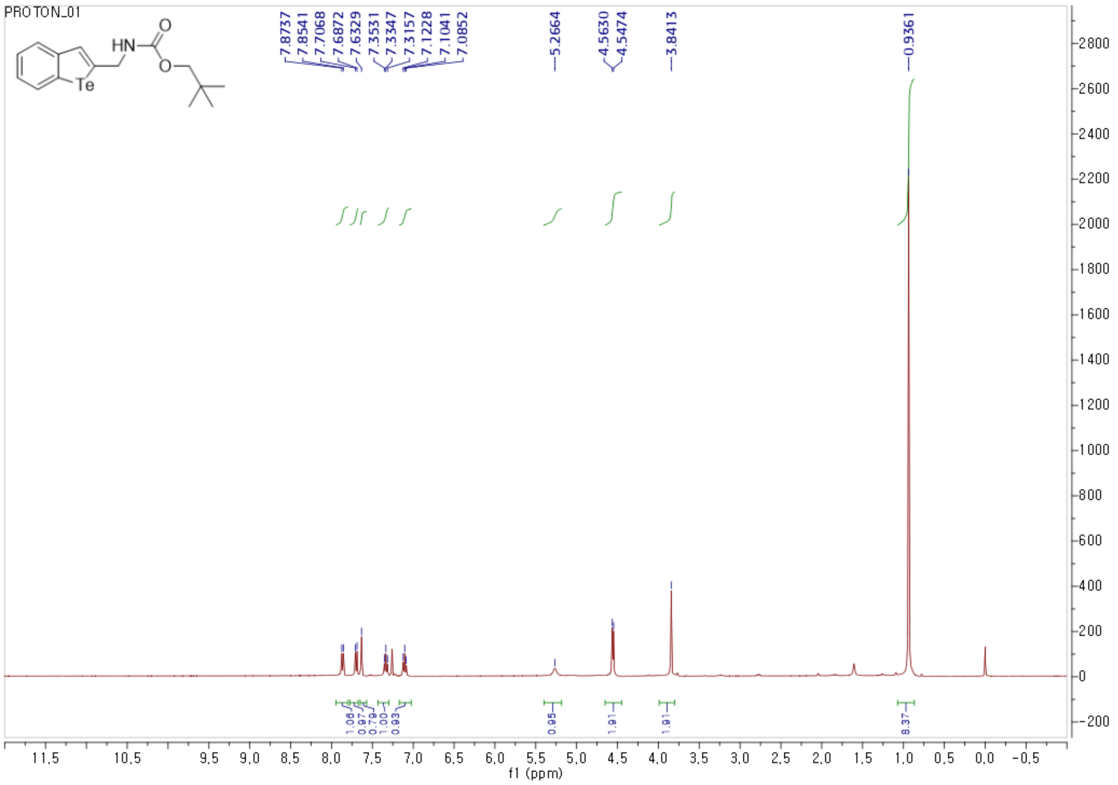


$^{13}$C NMR of **1g**

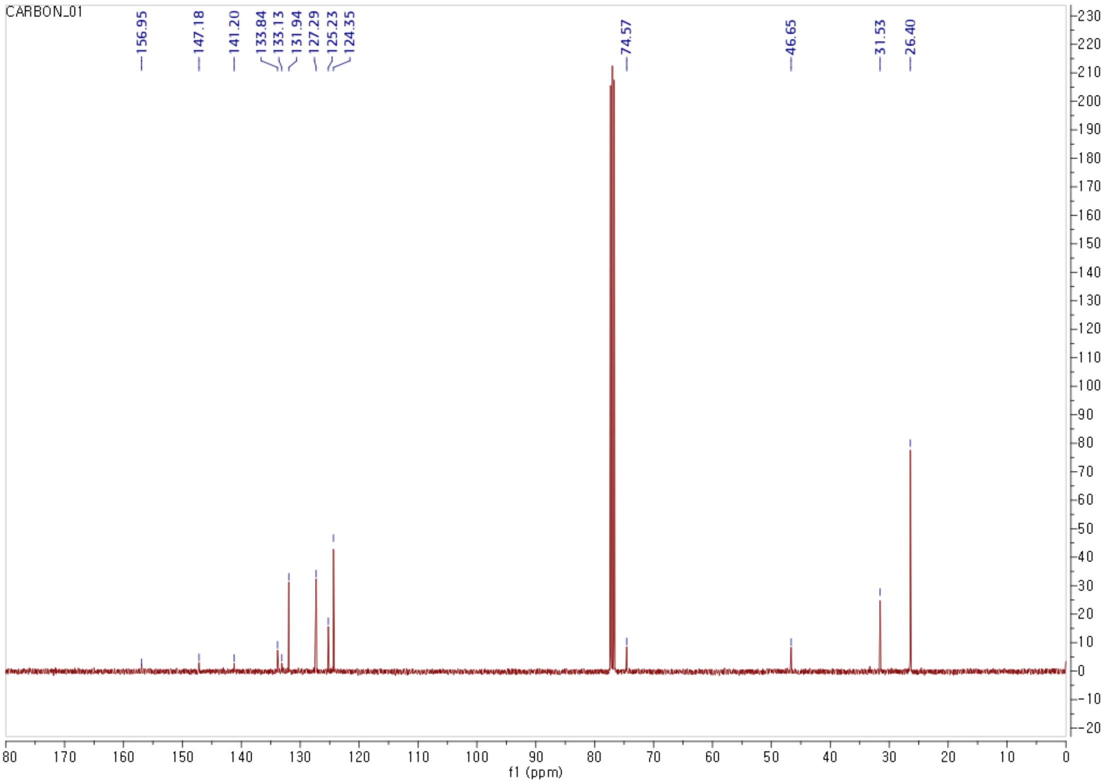

<sup></sup>

$^{1}$H NMR of **1h**

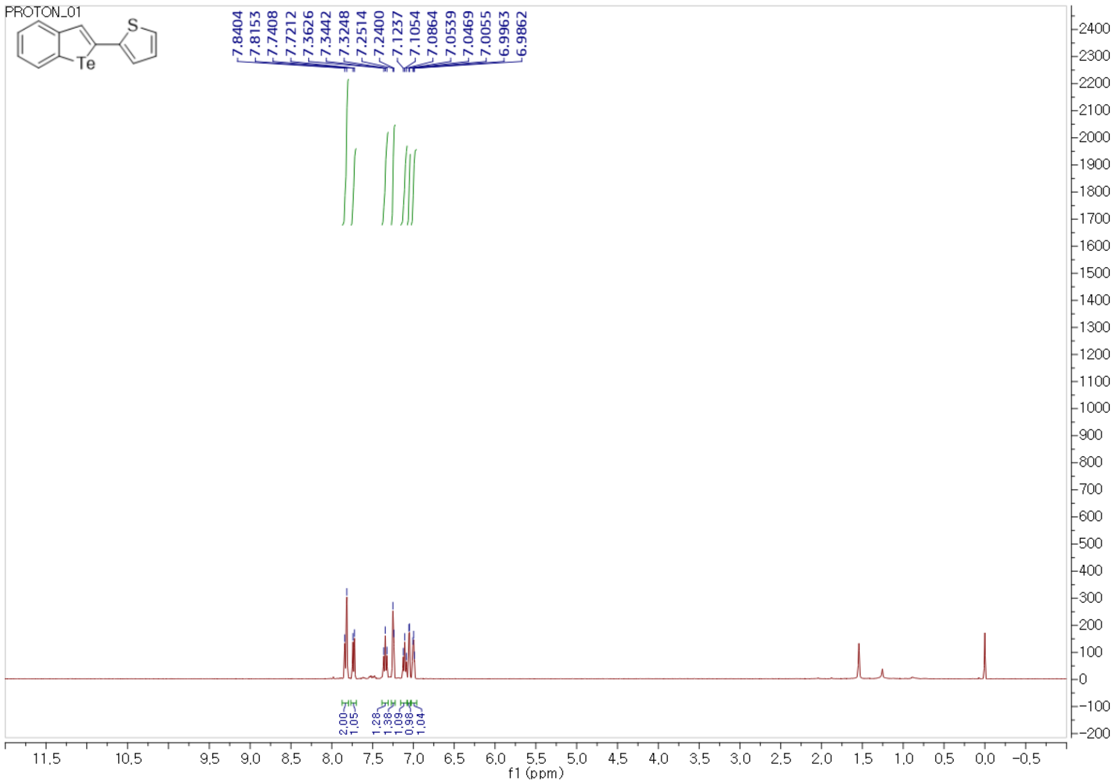

$^{13}$C NMR of **1h**

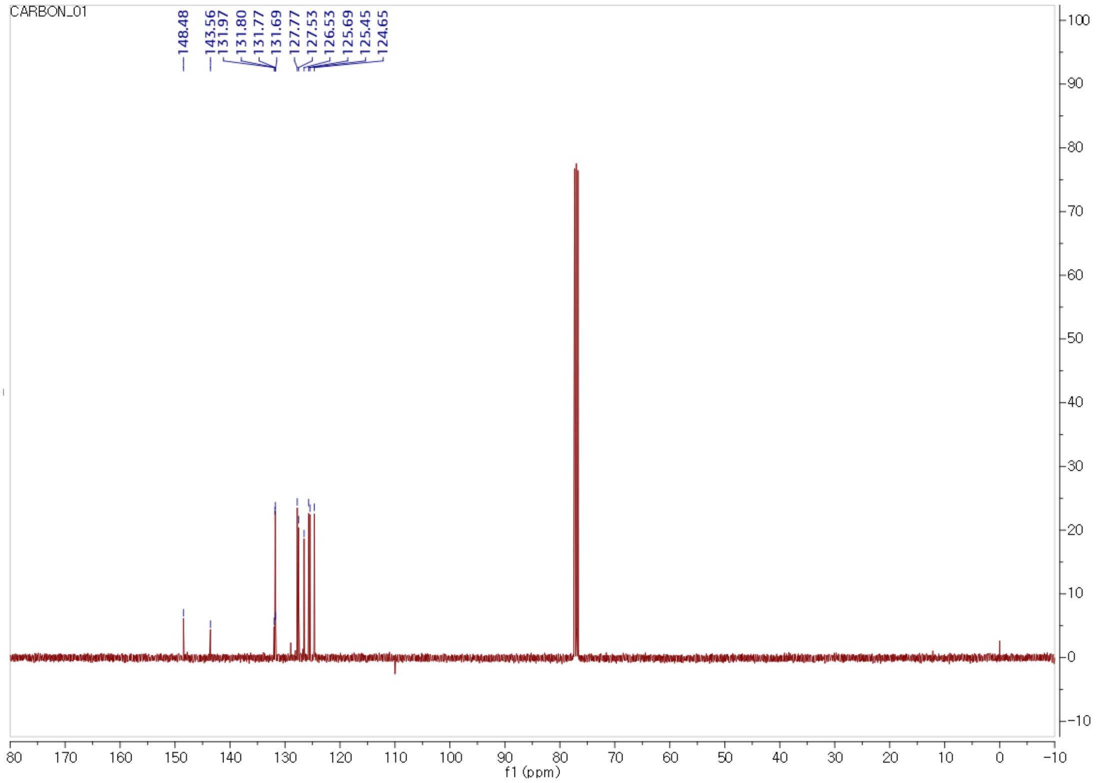

$^{1}H$ NMR of **1i**

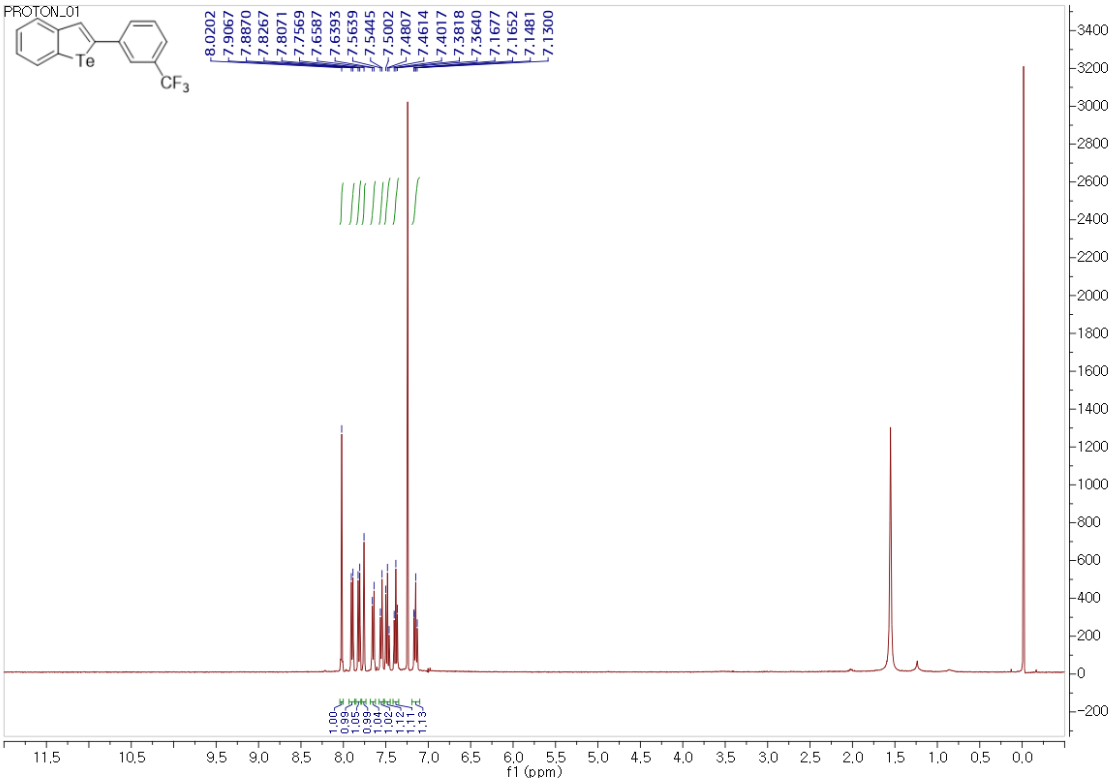


$^{13}C$ NMR of **1i**

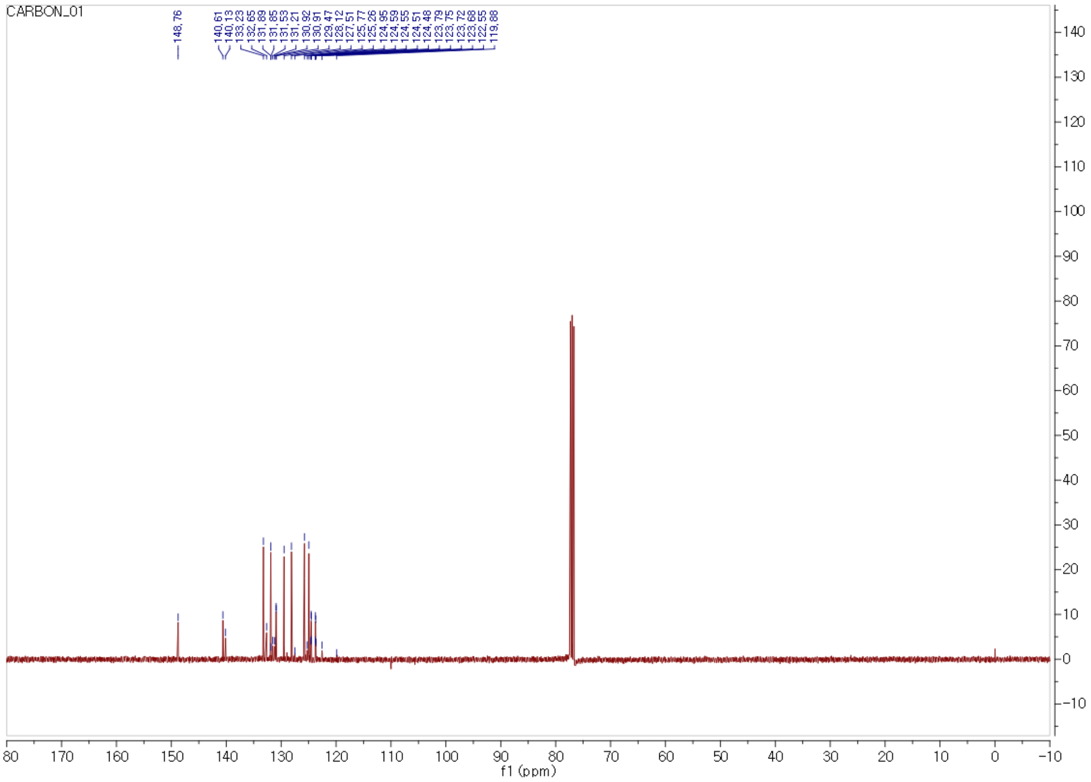

$^1$H NMR of **1j**

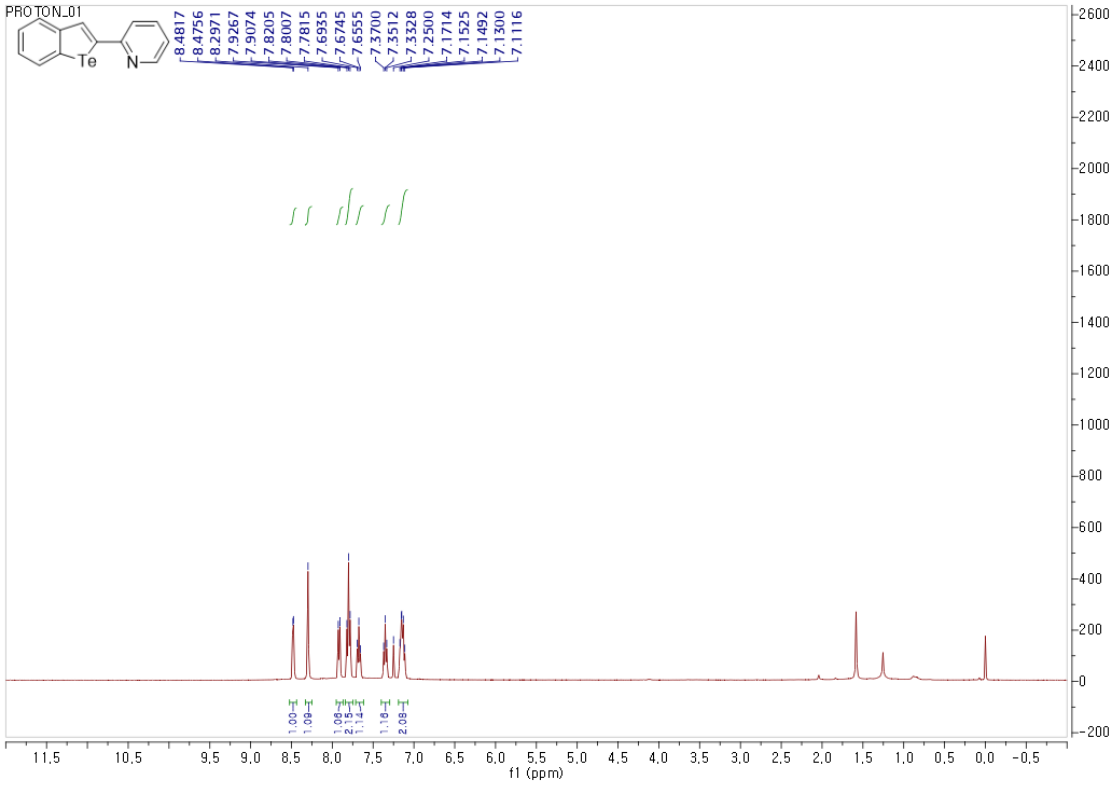


$^{13}$C NMR of **1j**

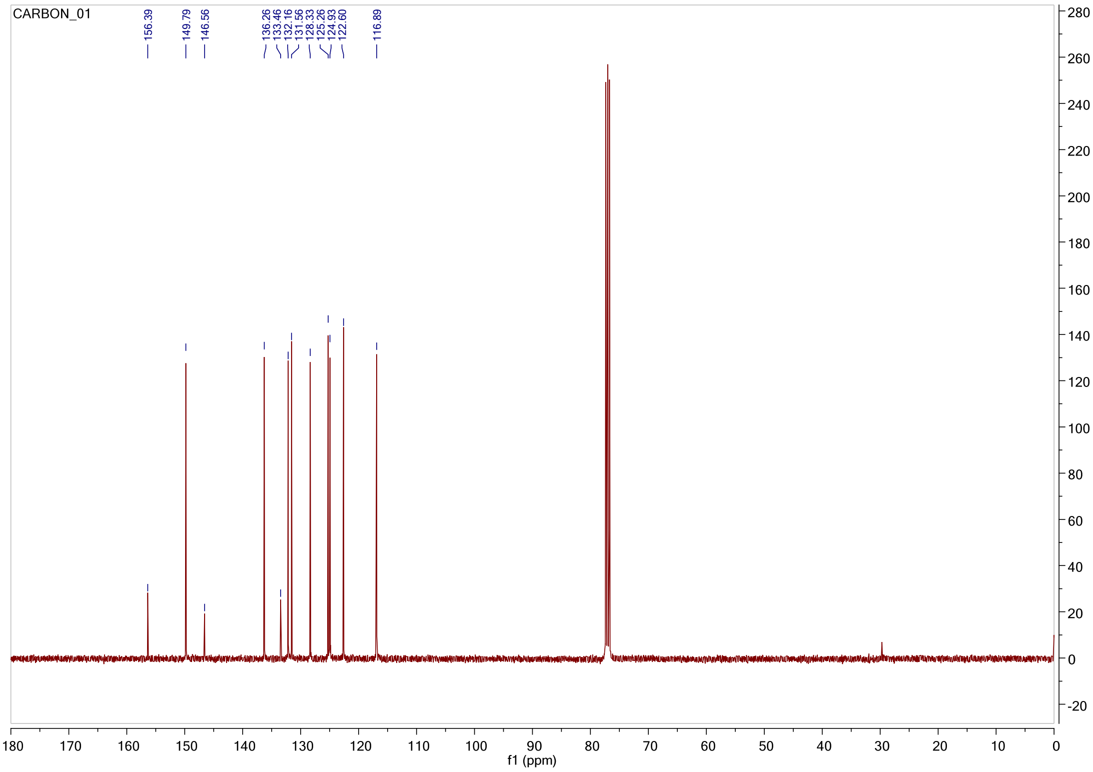

$^{1}$H NMR of **1k**

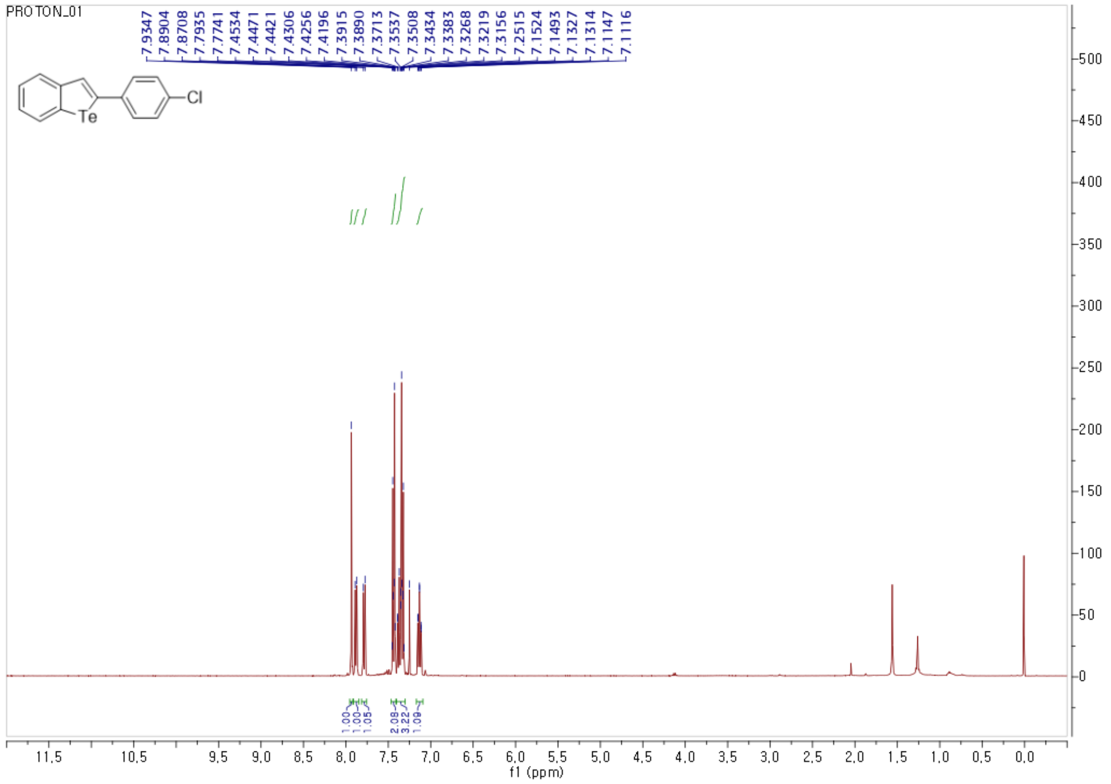


$^{13}$C NMR of **1k**

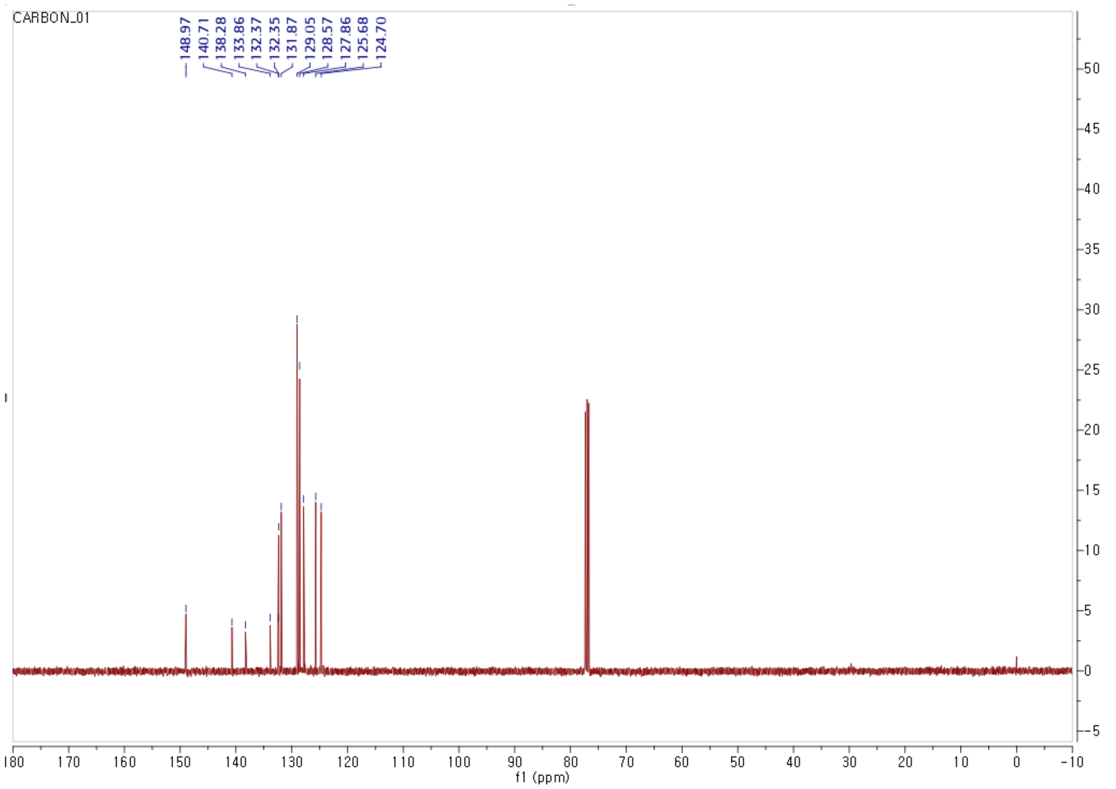

$^{1}$H NMR of **1l**

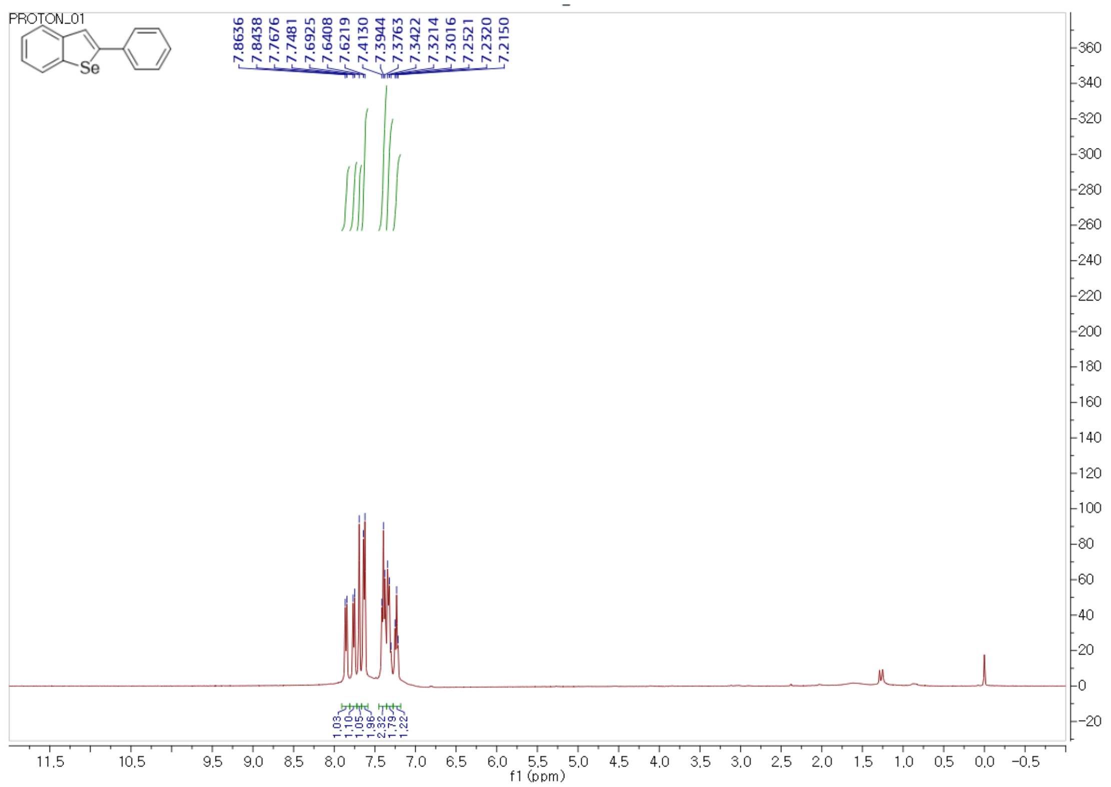


$^{13}$C NMR of **1l**

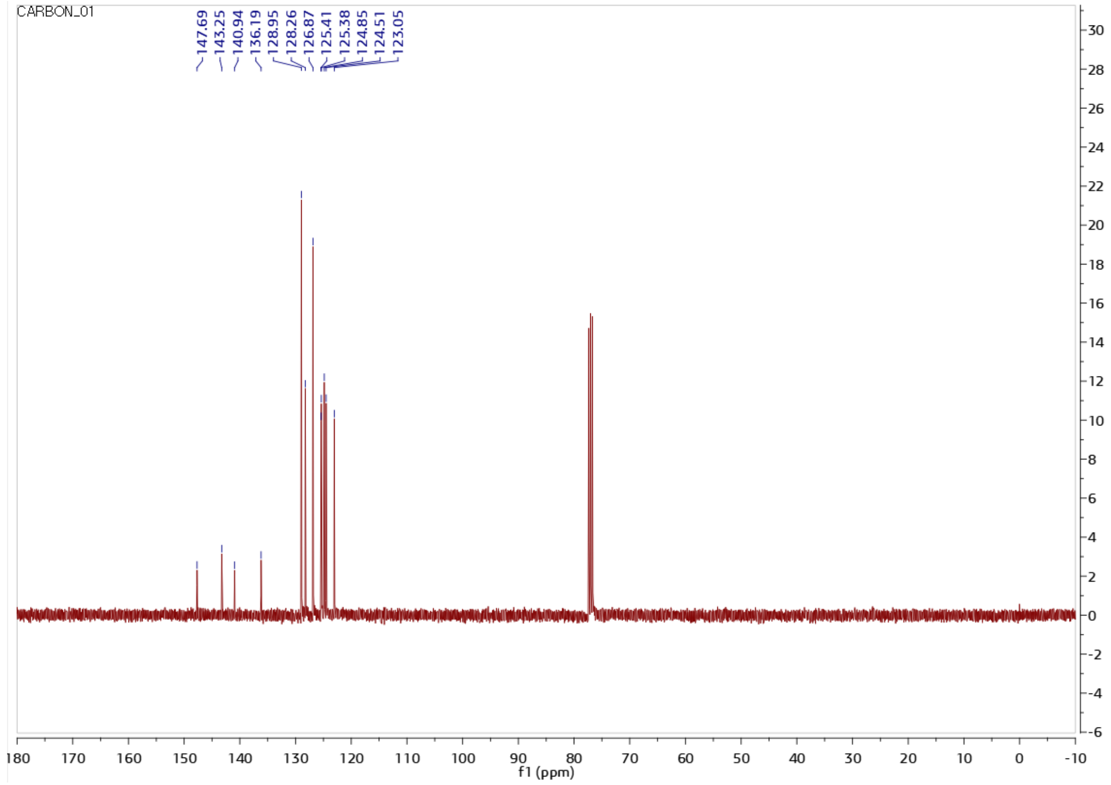

$^{1}$H NMR of **1m**

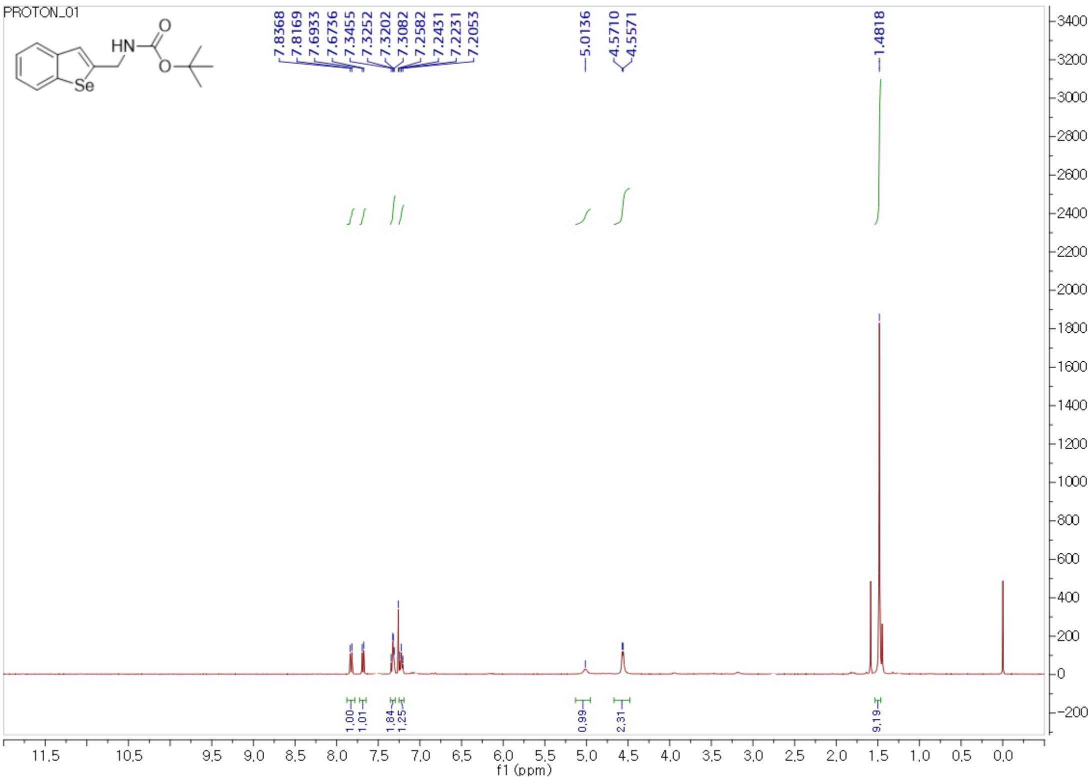


$^{13}$C NMR of **1m**

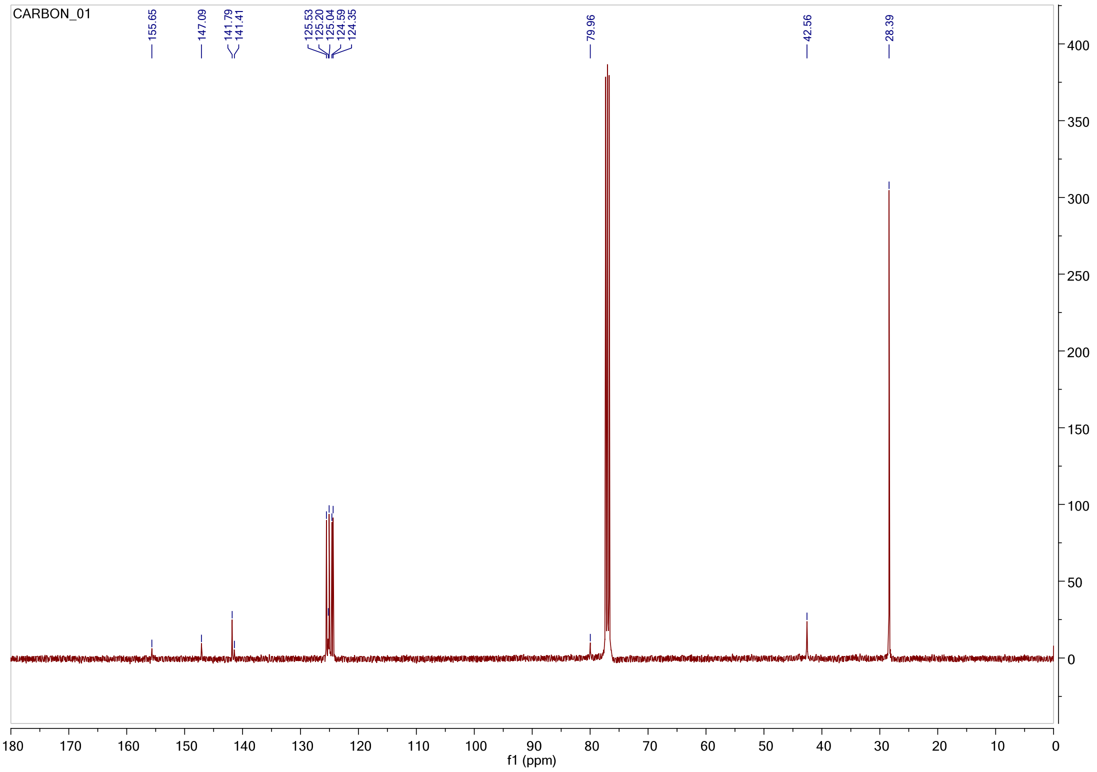

$^{1}$H NMR of **1n**

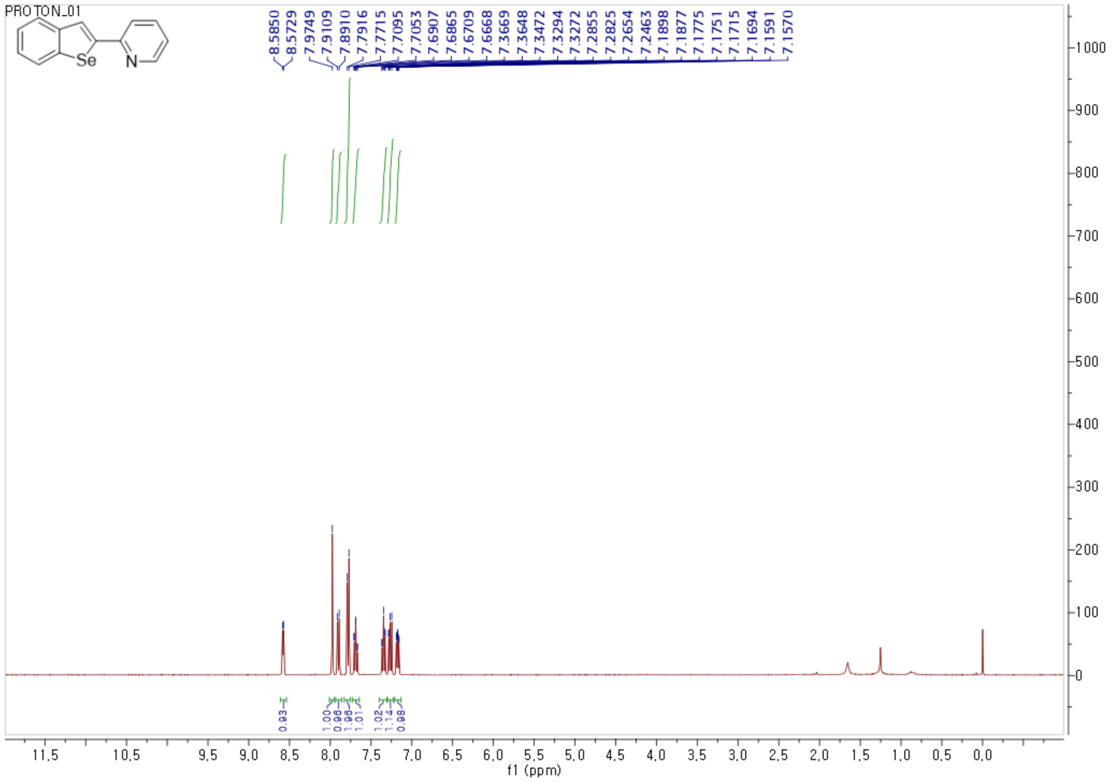


$^{13}$C NMR of **1n**

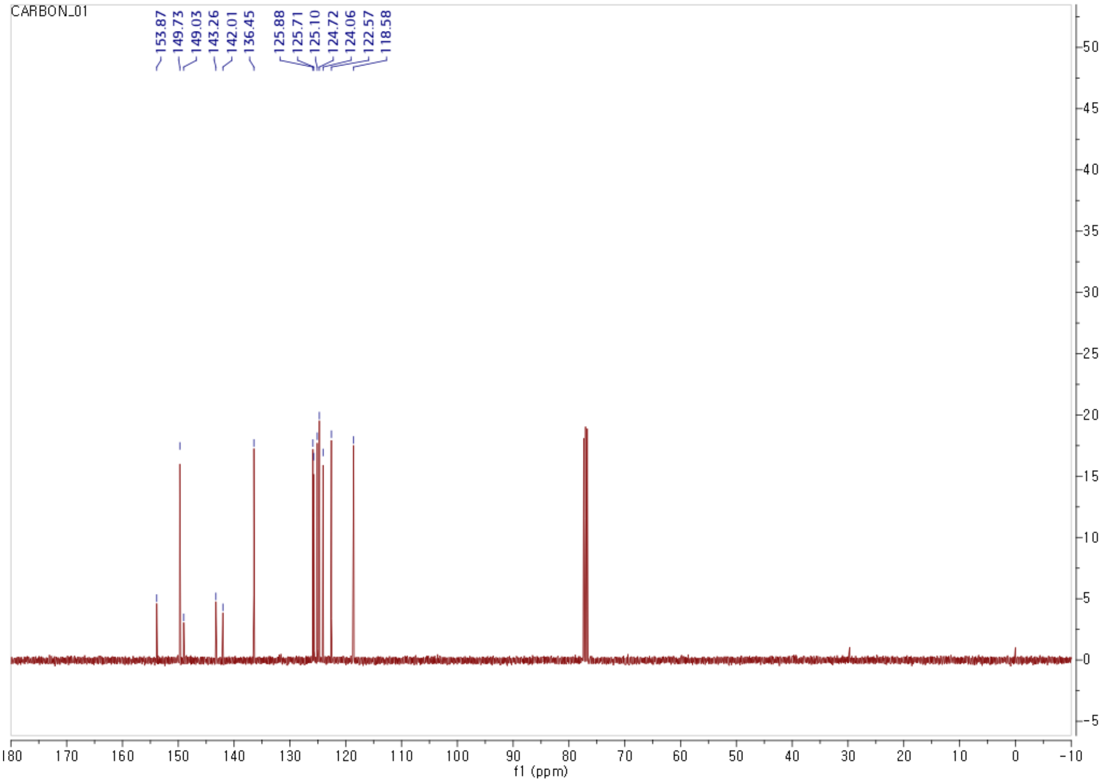

$^{1}$H NMR of **1o**

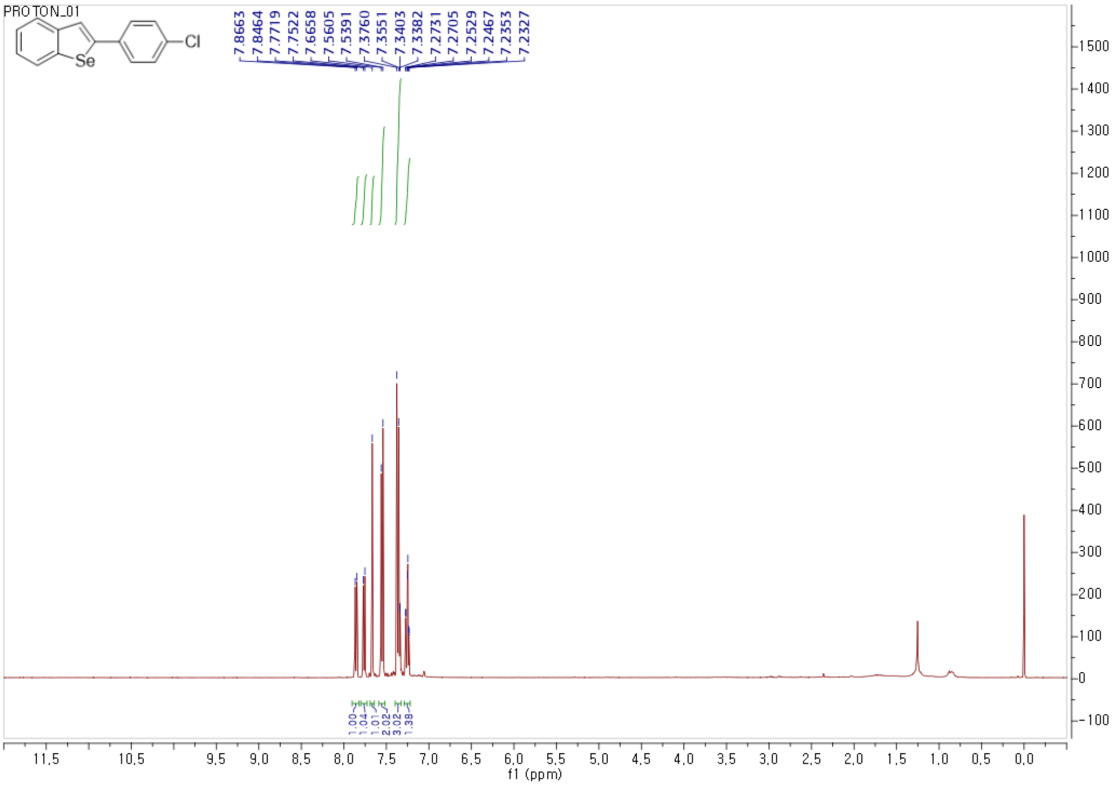


$^{13}$C NMR of **1o**

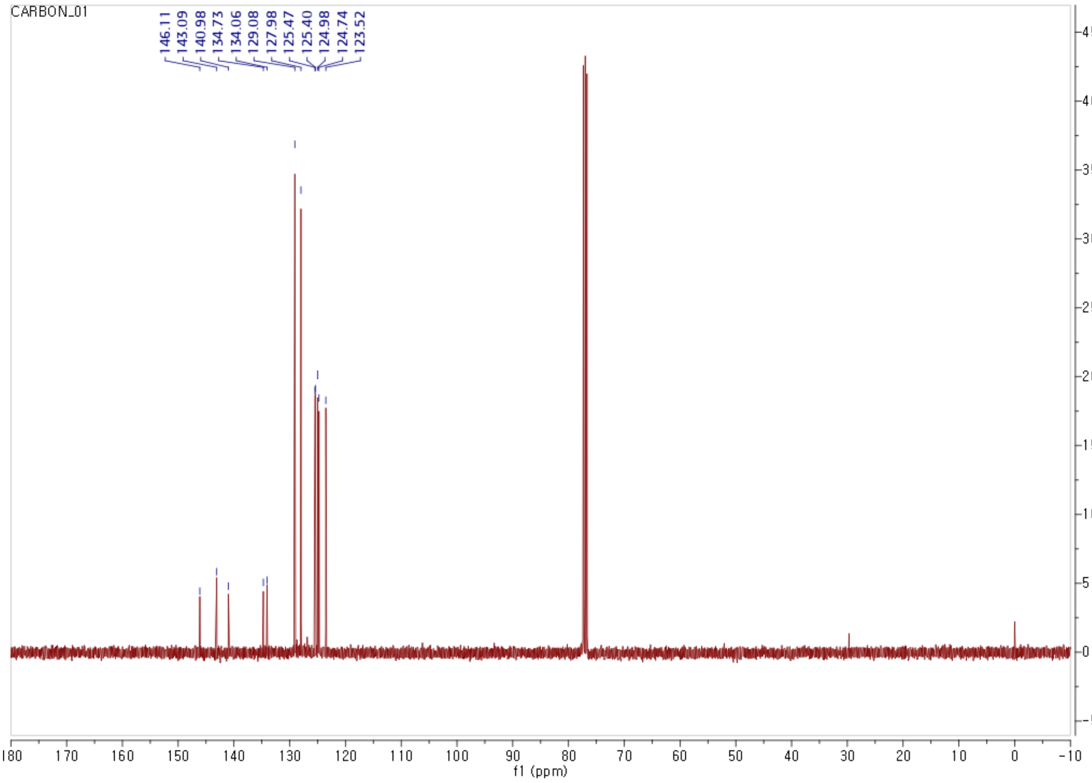

$^{1}$H NMR of **1p**

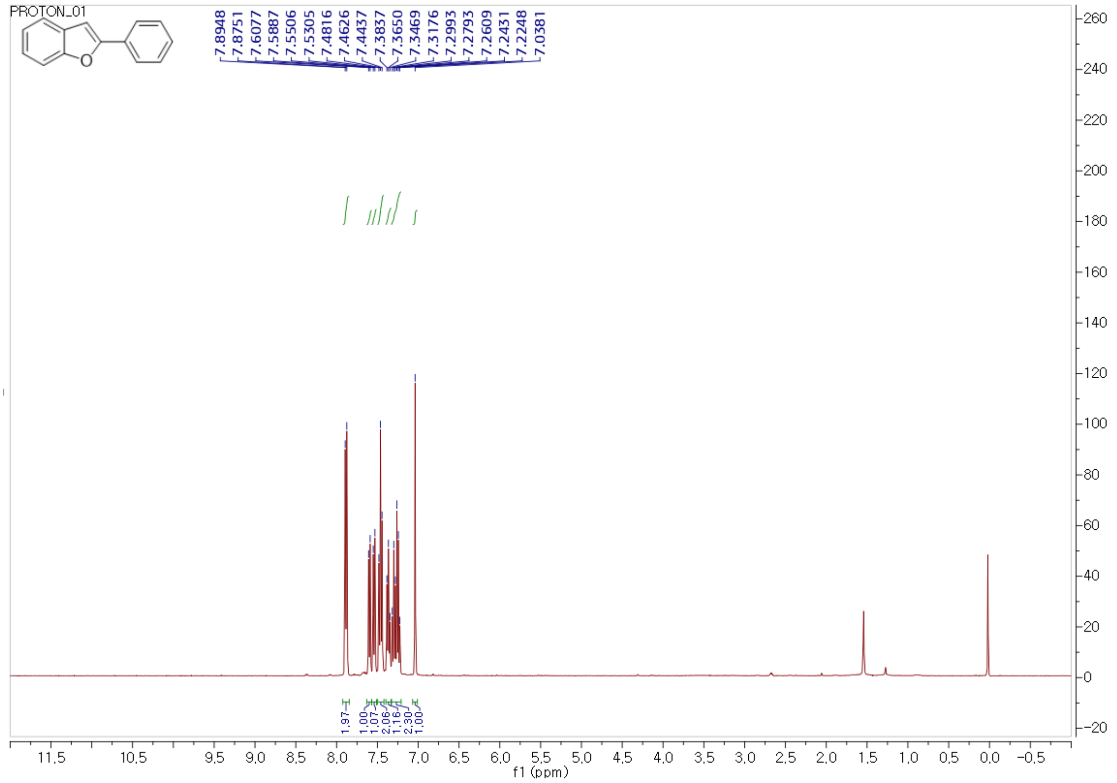


$^{13}$C NMR of **p**

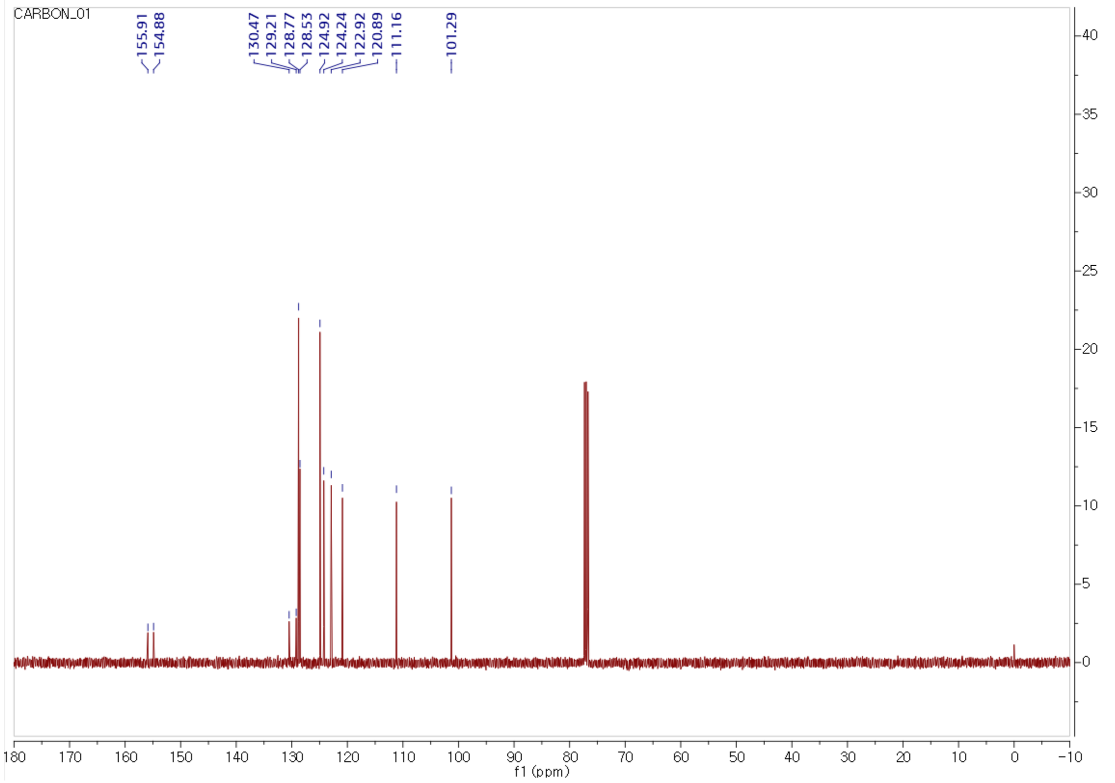

$^{1}$H NMR of **1q**

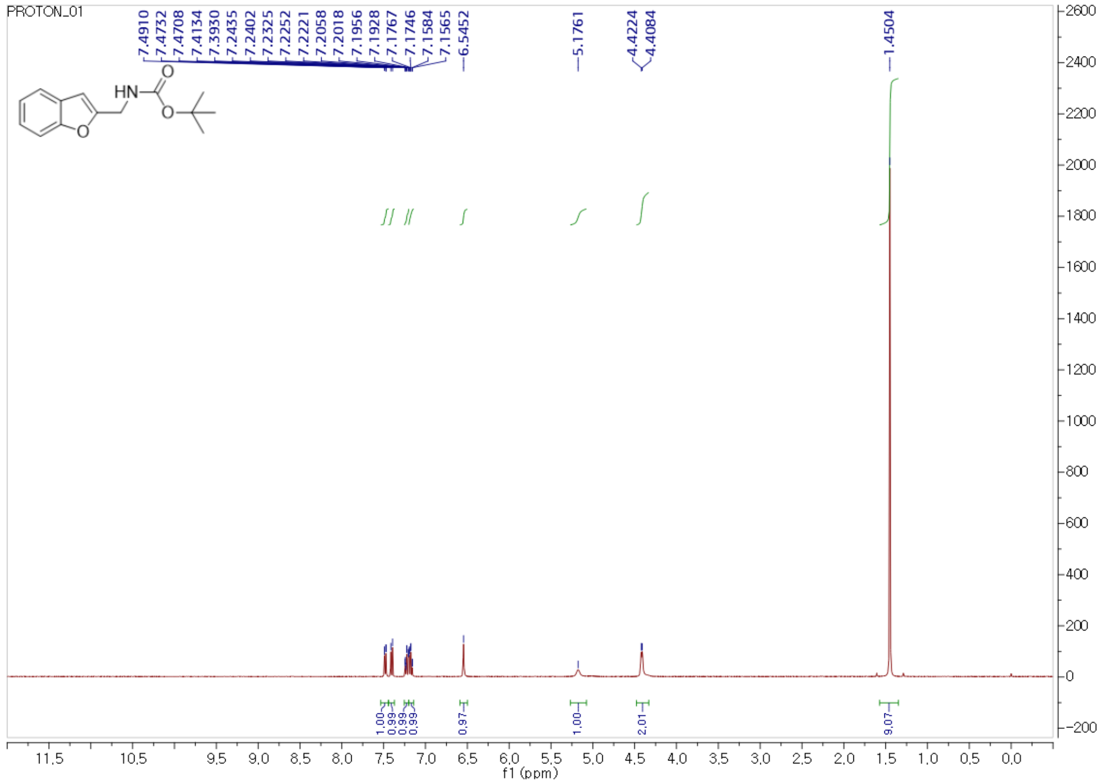


$^{13}$C NMR of **1q**

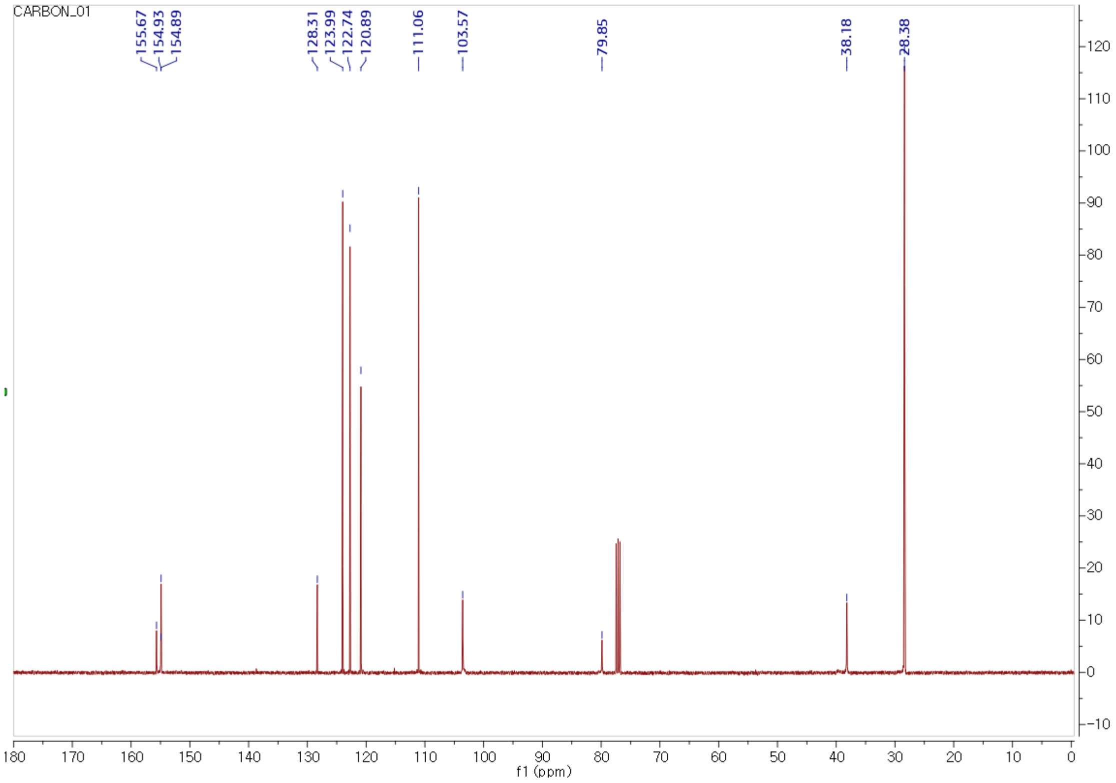

Supplement: Supplementary file 1 [file ijms-20-05908-s001.pdf]
